# Microbial Biomarkers of Intestinal Barrier Maturation in Preterm Infants

**DOI:** 10.3389/fmicb.2018.02755

**Published:** 2018-11-14

**Authors:** Bing Ma, Elias McComb, Pawel Gajer, Hongqiu Yang, Mike Humphrys, Adora C. Okogbule-Wonodi, Alessio Fasano, Jacques Ravel, Rose M Viscardi

**Affiliations:** ^1^Institute for Genome Sciences, Department of Microbiology and Immunology, University of Maryland School of Medicine, Baltimore, MD, United States; ^2^Department of Pediatrics and Child Health, Howard University College of Medicine, Washington, DC, United States; ^3^Department of Pediatrics, Basic, Clinical and Translational Research, MassGeneral Hospital for Children, Boston, MA, United States; ^4^Harvard Medical School, Boston, MA, United States; ^5^Department of Pediatrics, University of Maryland School of Medicine, Baltimore, MD, United States

**Keywords:** intestinal microbiota, preterm infant, necrotizing enterocolitis, leaky gut, intestinal permeability, Clostridiales, *Bifidobacterium*, breastmilk feeding

## Abstract

Intestinal barrier immaturity, or “leaky gut,” is the proximate cause of susceptibility to necrotizing enterocolitis in preterm neonates. However, the impact of intestinal microbiota development on intestinal mucosal barrier maturation has not been evaluated in this population. In this study, we investigated a longitudinally sampled cohort of 38 preterm infants < 33 weeks gestation monitored for intestinal permeability (IP) and fecal microbiota during the first 2 weeks of life. Rapid decrease in IP indicating intestinal barrier function maturation correlated with significant increase in community diversity. In particular, members of the Clostridiales and *Bifidobacterium* were highly transcriptionally active, and progressively increasing abundance in Clostridiales was significantly associated with decreased intestinal permeability. Further, neonatal factors previously identified to promote intestinal barrier maturation, including early exclusive breastmilk feeding and shorter duration antibiotic exposure, associate with the early colonization of the intestinal microbiota by members of the Clostridiales, which altogether are associated with improved intestinal barrier function in preterm infants.

## Introduction

The intestinal mucosa paracellular trafficking of macromolecules is controlled by a competent epithelial barrier (Lee, 2015). The intestinal barrier constitutes a protective shield to the diffusion of pathogens and other elements with pro-inflammatory and tissue injury properties, and regulates absorption and secretion of essential nutrients (Fasano, 2008). A functional intestinal barrier is driven by a complex structure that includes physical barrier with coinciding chemical, immunological and microbiological components (Neish, 2009). The colonization with microorganisms starts at birth and undergoes rapid shifts in composition and structure as the host matures over time (Koenig et al., 2011; Sharon et al., 2013; Grier et al., 2017; de Muinck and Trosvik, 2018). These microorganisms perform essential functions mechanistically linked to the immune system development, nutrient acquisition and energy regulation, opportunistic pathogens suppression, as well as intestinal barrier competency, which includes epithelial metabolism, proliferation and survival, mucin and antimicrobial compound production, and cell-cell communication signaling molecule secretion (Neish, 2009; Belkaid and Hand, 2014; Yu et al., 2016).

Disrupting intestinal microbiota, on the other hand, leads to dysbiosis, a state of ecological imbalance where the community loses diversity, key bacterial species, and more critically metabolic capacity with reduced colonization resistance to opportunistic pathogens (Arrieta et al., 2014). Early life gut dysbiosis is associated with disease susceptibility along with short-term and lifelong health issues, such as necrotizing enterocolitis (NEC) (Madan et al., 2012), sepsis (Madan et al., 2012), asthma and allergies (Arrieta et al., 2015), type 1 diabetes (Vatanen et al., 2016), celiac disease (Cenit et al., 2015), inflammatory bowel disease (Gevers et al., 2014) and obesity (Cho et al., 2012), among others. NEC is a life-threatening, gastrointestinal emergency affecting approximately 7–10% of preterm neonates with mortality as high as 30–50% (Guner et al., 2009). In this condition, bacteria cross the intestinal wall leading to local and systemic infection and inflammation, and bowel wall necrosis and perforation. Intestinal barrier immaturity, characterized as elevated intestinal permeability (IP), or “leaky gut,” is the proximate cause of susceptibility to NEC in preterm neonates (Anand et al., 2007; Fitzgibbons et al., 2009; Fox and Godavitarne, 2012; Bergmann et al., 2013). It is critical to characterize the preterm infant intestinal microbiota to identify dysbiotic states associated with increased intestinal leakiness, as well as beneficial bacteria associated with improved intestinal barrier function, for subsequent stratification of early diagnosis, early intervention and primary prevention of leaky gut and its sequelae.

Despite the critical role of the microbial community in intestinal barrier function, its effect on newborn IP is unknown. In particular, the microbiota of preterm neonates with measured elevated IP, a high-risk population for NEC, has not been previously studied. We hypothesize that the intestinal microbiota plays a pivotal role in modulating IP and that the presence of “beneficial” bacteria will be associated with improved intestinal barrier function in preterm infants. The percent urinary excretion of orally administered isotonic solutions of the non-metabolized sugars lactulose and rhamnose as markers of the intestinal paracellular and transcellular pathways, respectively, is the gold standard to measure IP and have been used extensively for over 30 years to assess IP in adults (Marchbank et al., 2008; van Wijck et al., 2012, 2013) and in preterm (Beach et al., 1982; Piena-Spoel et al., 2001; Rouwet et al., 2002; Albers et al., 2005; Corpeleijn et al., 2011; Westerbeek et al., 2011) and term infants (Piena et al., 1998; Albers et al., 2005; Malagon et al., 2005) as well as older children. IP was measured with coinciding measures of the composition and function of the fecal microbial communities were investigated. We sampled three time points during the first 2 weeks of life, which is a critical period corresponding to the initiation of the intestinal microbiota development process (Mackie et al., 1999; Mshvildadze et al., 2008; Saleem et al., 2017). A rapid decrease in IP was observed to correlate the increasing abundance of Clostridiales, indicating intestinal barrier function maturation over the first 2 weeks of life with a shift in the composition and structure in intestinal microbial community. Further, neonatal factors previously identified to promote intestinal barrier maturation, including early exclusive breastmilk feeding and less antibiotic exposure, associate with the early colonization of the intestinal microbiota by members of the Clostridiales, which altogether are associated with improved intestinal barrier function in preterm infants. Our results highlight the multifactorial processes involved in intestinal barrier maturation, and the importance to consider microbiological and neonatal factors for diagnosing, monitoring, and modulating IP in preterm infants in NEC prevention.

## Results

### Intestinal Barrier Maturation Correlates With Increased Microbiota Biodiversity Over the First Two Weeks of Life

**FIGURE 1:**
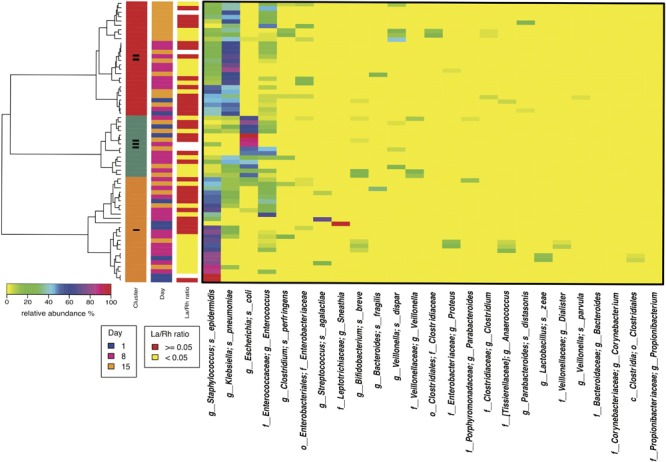
Heatmap of the 50 most abundant intestinal bacterial taxa relative abundance in samples collected from 38 preterm infants enrolled in the study. The microbiota of 64 fecal samples were successfully characterized by high-throughput sequencing of the V3–V4 variable regions of 16S rRNA genes. The three sidebars indicate cluster, time, and intestinal permeability category, respectively. Ward linkage clustering was used to cluster samples based on their Jensen-Shannon distance calculated in vegan package in R (Oksanen et al., 2011). The samples with no IP assessment were included to generated the clusters. The low and high intestinal permeability category was defined by a La/Rh > 0.05 or < = 0.05 respectively (Saleem et al., 2017). Taxonomic profiling of corresponding metagenomes further resolved *Klebsiella* spp. to *Klebsiella pneumoniae, Enterococcus* spp. to *Enterococcus faecalis*, and *Bifidobacterium* spp. to *Bifidobacterium breve*.

**FIGURE 2:**
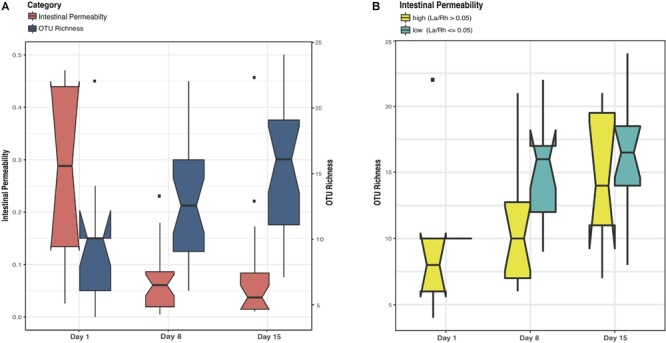
Boxplots comparing levels of intestinal permeability and microbial community diversity at study days 1, 8, and 15 in a cohort of 38 preterm infants (<33 weeks gestational age). Intestinal permeability is measured by non-metabolized sugar probes lactulose (La) (marker of intestinal paracellular transport)/rhamnose (Rh) (marker of intestinal transcellular transport). Microbial community diversity was calculated by OTU (Operational Taxonomic Units) richness. Wilcoxon rank sum test and a false discovery rate of 5% was used in significance test. Median values and interquartile of the values were shown in box. **(A)** Intestinal permeability (*p*-value = 0.002) and community diversity at the three study time points (*p*-value = 0.02). **(B)** Community diversity (*p*-value < 0.001) in infants with low and high intestinal permeability defined by a La/Rh > 0.05 or ≤0.05 respectively (Saleem et al., 2017).

Study days were defined as day 1, day 8 ± 2 and day 15 ± 2 with the first study day within 4 days after birth. Off 43 subjects < 33 weeks gestation who were enrolled in the study, stools samples were available from 38. The demographic, obstetric, and neonatal characteristics for included subjects are summarized in Table 1 and listed in Supplementary File S3. The study cohort has on average birthweight at 1,386 ± 404 g and gestational age at 29.9 ± 2.2 weeks. The mean IP measurement of this cohort including all timepoints is 0.10 ± 0.12 and 52.5% of the sampling points have elevated IP. The microbiota of 64 fecal samples were successfully characterized by high-throughput sequencing of the V3–V4 variable regions of 16S rRNA genes. A total of 422,444 high-quality amplicon sequences was obtained, corresponding to 10,544 (±4,029) sequences per sample with an average length of 428 bp. The top 25 most abundant phylotypes are shown in Figure 1. Taxonomic profiles of all samples were clustered into three distinct groups according to similarities in community composition and structure. *Klebsiella* spp., *Staphylococcus epidermidis*, and *Escherichia coli* dominated cluster I, II, and III, respectively. Both La/Rh ratio and taxonomic profile of each sample are shown in Supplementary File S3. Taxonomic profiling of corresponding metagenomes further resolved *Klebsiella* spp. to *Klebsiella pneumoniae, Enterococcus* spp. to *Enterococcus faecalis*, and *Bifidobacterium* spp. to *Bifidobacterium breve*. Taxonomic profiles of stool samples from infants at 6–24 months of age born at term, or phase II/III as defined previously (Mackie et al., 1999; Mshvildadze et al., 2008; Unger et al., 2015), clustered together in a different and more diverse cluster (Supplementary Figure S1). Rapid decrease in IP over the 2-week observation period indicates intestinal barrier function maturation (*p*-value = 0.002), which is correlated with a significant increase in community diversity (*p*-value = 0.02) (Figure 2A); while delayed increase in community diversity was associated with persistence of high intestinal permeability (*p*-value < 0.001) (Figure 2B). The results indicated that preterm infant intestinal barrier maturation correlates with increased fecal microbiota biodiversity and a change in microbiota composition and structure.

**Table 1:** Characteristics of study subjects (preterm infants < 33 weeks gestational age).

	*N*	%
**Ethnicity**		
African American	22	57.9
Asian	3	7.9
White	12	31.6
Others	1	2.6
**Gender**		
Female	17	44.7
Male	21	55.3
**Delivery route**		
Cesarean	26	68.4
Vaginal	12	31.6
**Gestational age/Postmenstrual age^∗^ (weeks)**	29.9 ± 2.2/31.4 ± 2.3	
**Birth weight**	1,386 ± 404	
<1500 g	21	55.3
≥1500 g	17	44.7
**Intestinal permeability ^∗∗^**	0.104 ± 0.123	
High	31	52.5
Low	28	47.5
**Antibiotic use**		
None	7	18.4
1–3 days	12	31.6
>4 days	19	50.0
**Day start breastmilk feeding**		
Day 1	17	44.7
Day 2 or 3	15	39.5
>Day 4	6	15.8
**Day reached full breastmilk feeding**		
<Day 7	5	13.2
Day 8–14	15	39.5
>Day 15	18	47.4
**Microbiota type ^∗∗∗^ (most abundant species)**		
I *(Klebsiella pneumonia)*	27	42.2
II *(Staphylococcus epidermidis)*	26	40.6
III *(Escherichia coli)*	11	17.2

*^∗^Postmenstrual age was calculated as gestational age at birth plus week of life (Grier et at., 2017). ^∗∗^The low and high intestinal permeability category was defined by a La/Rh > 0.05 or ≤0.05, respectively (Saleem et al., 2017). ^∗∗∗^Microbiota type was defined based on clustering of taxonomic profiles using 16S rRNA gene amplicon in this study.*

### Subject Variation, Postmenstrual Age, and IP Explain Most of the Variation in Intestinal Microbiota

We employed multivariate response linear regression on the “balance” of microbial community and evaluated the effect of covariates of demographic, obstetric, and neonatal factors on the microbiome using Gneiss (Morton et al., 2017). Covariates of antibiotics use, maternal antibiotics use, delivery mode, preterm premature rupture of membranes (pPROM), feeding pattern, IP, birthweight, gender, ethnicity, gestational age (GA) and postmenstrual age (PMA) were included in the analysis. Subject, PMA, and IP had the greatest correlation with intestinal microbiota, together they explained 63.4% of the variation of the intestinal microbial community composition observed in the cohort (Supplementary File S4). The predicted points lie within the same region as the original communities and the residuals have roughly the same variance as the predictions within ±2, indicating the model is fitting the data well (Supplementary Figure S2). Overall our result indicates the microbial differences between subjects are large (R squared difference is 0.023 ± 0.01), and the covariate with strongest effect is PMA (*R* squared difference is 0.044), suggesting that intestinal microbial composition has subject-to-subject difference and is development-dependent. IP correlates with the intestinal microbiota (*R* squared difference is 0.020), and its effect is lower than PMA and similar to the average among-subject effect.

### Clostridiales Is Associated With Low Intestinal Permeability in Preterm Infants

**FIGURE 3:**
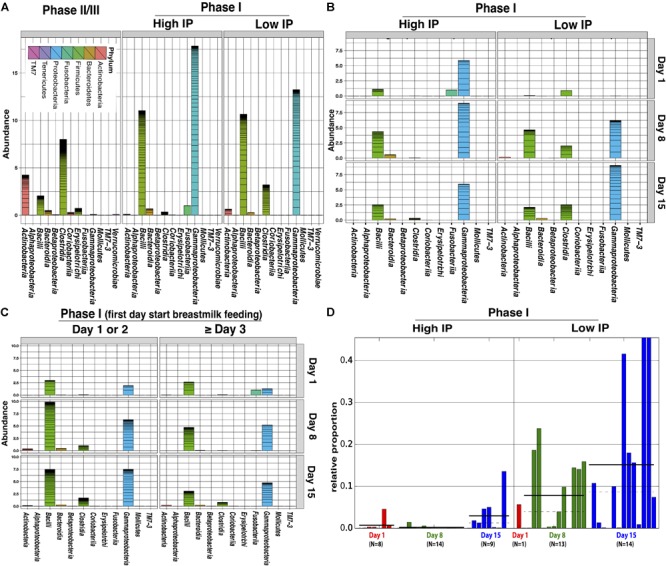
Comparison of relative abundance of bacterial groups in stool samples associated with high and low IP (La/Rh) measurements. **(A)** Cumulative abundance between phase II/III subjects (6–24 months of age) and phase I infants (within first 2 weeks of life) with high and low IP; **(B)** Cumulative abundance at study day 1, 8, and 15 for phase I infants with high and low IP. **(C)** Cumulative abundance at study day 1, 8, and 15 for phase I infants that had first day starting breastmilk feeding at day 1 or 2, or day 3 and later. **(D)** The relative abundance of *Clostridiales* of each sampling point and the number of samples at study day 1, 8, and 15 for phase I infants with high or low IP. Bars represent the relative abundance of *Clostridiales* in each sample. Dotted line and solid line represent mean, and median relative abundance, respectively. *Clostridiales* was identified to be significantly discriminative with respect to the IP class [*p*-value = 0.0002, logarithmic linear discriminant analysis (LDA) score is 4.996] using LDA effect size (LEfSe) analysis (Segata et al., 2011). The alpha threshold value for the pairwise non-parametric Kruskal–Wallis test was 0.05 and the threshold for the logarithmic LDA model score (Fisher, 1936) for discriminative features was 2.0. An all-against-all comparison in multi-class analysis was performed. The low and high IP category was defined by a La/Rh > 0.05 or ≤0.05 respectively.

Comparative analysis of fecal microbiota with high (La/Rh ≥ 0.05) and low IP (La/Rh < 0.05) showed that *Clostridia*, the class containing the only order Clostridiales in this cohort, was significantly more abundant in samples with low IP compared to those with high IP (*p*-value = 0.01) (Figures 3A,B and Supplementary Figure S3). In particular, a progressive and significant increase in members of Clostridiales over the first 2 week after birth significantly associated with low IP (*p*-value = 0.0002) as shown in Figure 3D. The significance level was calculated based on all samples from the three time points. Based on Bayesian non-parametric adaptive smoothing models and subject-specific changes in relative abundance of Clostridiales at study day 1, 8, and 15, the results demonstrated: (1) at baseline study day 1 within 4 days of birth, the abundance of Clostridiales was low in subjects with either high or low IP; (2) However, in samples measures with low IP but not high IP, a significant increase in Clostridiales was observed that reached ∼8% median and >20% maximal relative abundance at study day 8, and ∼16% median and ∼45% maximal relative abundance at study day 15; (3) on the other hand, in infants with persistently high IP (delayed maturation), members of Clostridiales was almost completely absent on study day 8 and no increase was observed from study day 1 to 8, and its abundance level on study day 15 was only at ∼3% median and ∼10% maximal relative abundance; (4) in infants 6–24 months old, Clostridiales is the most abundant taxonomic groups with >50% median and >85% maximal relative abundance (Supplementary Figure S4A). Interestingly, for the subjects that started breastmilk feeding within the first 1 or 2 days, it also appears Clostridiales was progressively more abundant at the end of the first and second weeks. On the contrary for the subjects that started breastmilk feeding on study day 3 or later, the Clostridiales were less established (Figure 3C), highlighting the impact of early breastmilk feeding on bacterial colonization that shapes intestinal community. Together, our results suggest preterm infants at birth have low abundance of Clostridiales, which became progressively and significantly more abundant only in the group with rapid progression of intestinal barrier maturation, while remained low in those with persistent high IP over the first 2 weeks of life.

We further calculated the predictive power of microbiota composition in classifying IP using random forest supervised machine learning scheme. The top 15 phylotypes with the highest mean decrease gini index importance measure (Supplementary Figure S5) were used to fit a random effect logistic regression model of IP, 4 of which were significantly associated with low IP (Supplementary File S5), including three members of the order Clostridiales, *Coprococcus* (*p*-value = 0.004), *Lachnospiraceae* (*p*-value = 0.007), *Veillonella dispar* (*p*-value = 0.01), and *Bifidobacterium* (*p*-value = 0.01) from the order of Bifidobacteriales. Interestingly, Bifidobacteriales was the second most abundant taxonomic groups in infants 6–24 months old, only lower than Clostridiales (Supplementary Figure 4B).

### Clostridiales and *Bifidobacterium* Are Highly Active Members of the Intestinal Microbiome

**FIGURE 4:**
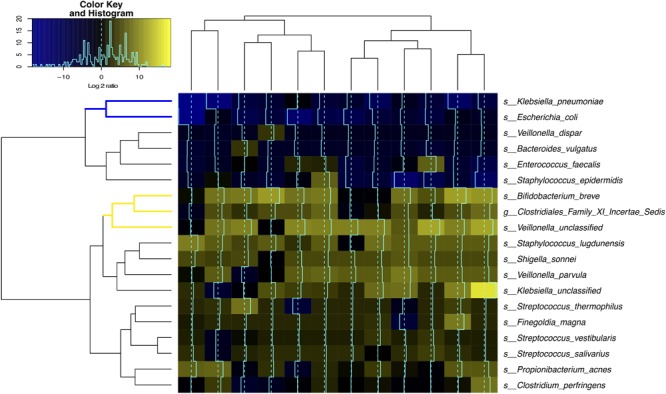
Bacterial species transcriptional activity in preterm infant stools. Fecal samples are represented in columns and taxonomic composition quantified using MetaPhlAn (Segata et al., 2012) version 2 are shown in rows, both are organized by hierarchical clustering. Normalization using Witten-Bell smoothing was performed, and the relative expression of a gene in a sample was calculated by normalizing the smoothed value of the expression level in the metatranscriptome by the smoothed value of the corresponding gene abundance in the metagenome (Franzosa et al., 2014; Franzosa et al., 2015). Color scheme indicates an approximate measure of the species’ clade-specific transcriptional activity (Franzosa et al., 2014). The colored branches show the clustering of bacterial species that are consistently transcriptionally active (yellow) or consistently transcriptionally inactive (blue) across samples.

The level of bacterial transcriptional activities was characterized by studying the suite of genes present and expressed in preterm infant intestinal microbiota. A total of 869 million metagenomic sequence reads (average of ∼31.0 million sequence reads per sample) and 694 million metatranscriptomic sequence reads (average of ∼53.4 million sequence reads per sample) were obtained after quality assessment. Figure 4 shows that *Bifidobacterium breve* (Actinobacteria), *Veillonella* and *Clostridiales Family XI incerteae Sedis* (Clostridiales) were the most transcriptionally active bacteria with high ratio of transcript abundances over gene abundances in all samples. Further, the levels of transcriptional activities of *Bifidobacterium breve* and *Clostridiales Family XI incerteae Sedis* were correlated with a spearman correlation of 0.89, suggesting these two taxonomic groups are either functionally dependent or co-regulated (Supplementary Figure S6). We observed increased abundance of both Clostridiales and *Bifidobacteriales* through the transition from the first 2 weeks (phase I) to later age of 6–24 months (phase II/III) as further supporting their active contribution to the function of the GI microbiota after birth. Interestingly, Clostridiales and Bifidobacteriales were also the most abundant taxonomic groups in the intestinal microbiota of 6–24 months old infants (Supplementary Figures S4A,B and Supplementary File S3). Specifically, members of the family Clostridiales have an average abundance of 50 ± 3% in phase II and III infants, compared to 0.1 ± 0.4% in phase I infants. Bifidobacteriales have an abundance of 26 ± 5% in phase II and III as opposed to 0.1 ± 0.3% in phase I infants. Together with the previous observation that *Coprococcus* (Clostridiales), *Lachnospiraceae* (Clostridiales), *Veillonella dispar* (Clostridiales), and *Bifidobacterium* (Bifidobacteriales) are significantly associated with low IP, our results suggest the presence and more importantly the activity of bacterial members of Clostridiales and Bifidobacteriales are associated with improved intestinal barrier maturation.

Conversely, the two *Enterobacteriaceae* species, *Klebsiella pneumoniae* and *Escherichia coli*, had low transcriptional activity despite their high relative abundance in the infant GI microbiota, questioning their functional contribution to the infant stool microbiota. Interestingly, *Enterobacteriaceae* and *Staphylococcus* are the most abundant bacterial taxa present in phase I infants but are rarely observed in phase II and III (Supplementary Figures S4C,D).

### Early Breast Milk Feeding and Shorter Duration Antibiotic Exposure Positively Correlates With Clostridiales Abundance and Activities

The associations between intestinal microbiota and demographic, obstetric, and neonatal factors were also evaluated. Gneiss analysis suggests delivery mode, pPROM, gender, ethnicity, birthweight, maternal antibiotics use are not contributing covariates to the intestinal microbial community variance. Further, no bacterial phylotype was identified to significantly associate with these factors. However, breast milk feeding pattern and shorter duration infant antibiotic exposure were significantly associated with increased abundance of *Clostridiales.* More specifically, early full exclusive breast milk feeding by study day 10 (*p*-value = 0.0001) (Supplementary Figure S7) and antibiotic exposure limited to no more than 4 days (*p*-value = 0.05), were associated with the family *Lachnospiraceae* in the Clostridiales (*p*-value = 0.004) (Supplementary Figure S8). On the other hand, *Enterobacteriaceae*, particularly *Klebsiella pneumoniae* (as identified by metagenomics sequencing), was significantly associated with full breast milk feeding achieved after study day 10 (*p*-value = 0.01) (Supplementary Figure S7). These results strongly suggest members of the Clostridiales are significantly associated with low intestinal permeability, early full breast milk feeding, as well as shorter duration of antibiotic use.

### Clostridiales Are Highly Prevalent in the GI Microbiota of Preterm Infants

The most abundant bacterial species that included *K. pneumoniae, Staphylococcus epidermidis, E. coli*, and *Enterococcus faecalis* were found with mean abundance of ∼10–35% (S.D. ∼15–30%) and ∼85–95% prevalence in these samples. In comparison, many species such as *Streptococcus agalactiae, B. breve, B. longum, Clostridium perfringens, Propionibacterium acnes, Bacteroides fragilis, Veillonella parvula*, and *Streptococcus thermophiles* were present in 15–70% of all samples and had a much lower level of abundance ranging from ∼0.0001–1% (S.D. ∼0.0001–6%). Many of the members of Clostridiales were not resolved at the species or genus-level, while those taxonomically identified Clostridiales included *Coprococcus, Blautia, SMB53, Ruminococcus gnavus, Clostridium* spp., *Faecalibacterium prausnitzii, Dorea, Ruminococcus bromii, Roseburia, Pseudoramibacter* and *Butyricicoccus pullicaecorum* were detected in low or extremely low abundance yet high prevalence (Supplementary File S3). Given our average sequencing depth is ∼10^4^–10^5^ and stool bacterial load per gram of stool is in the range of ∼10^6^–10^10^ (Palmer et al., 2007; Abdulkadir et al., 2016; Wandro et al., 2018), it is likely that some bacterial taxonomic groups with low relative abundance (0.0001–1%) are below our detection limit. It is expected that the prevalence of the members of Clostridiales can be underestimated that is actually higher than the currently observed 15–70% among samples in the GI microbiota of preterm infants.

## Discussion

Preterm infants are at elevated risk for leaky gut, feeding intolerance, NEC and sepsis, and other short-term and long-terms morbidities (Unger et al., 2015). The pathophysiology of these disorders is likely multifactorial, involving a combination of intestinal mucosa barrier immaturity, imbalance in microvascular tone, aberrant microbial colonization and altered immune responses (Mai et al., 2011; Neu and Walker, 2011; Unger et al., 2015). Our group and others previously demonstrated that neonatal factors such as gestational age, antibiotic exposure, and exclusive breastmilk feeding affect intestinal mucosa barrier permeability in preterm infants (Taylor et al., 2009; Saleem et al., 2017). With the rapid development of high-throughput sequencing technology, recent studies have evaluated the significant association between the composition of intestinal microbiota, neonatal intestinal health and development (Palmer et al., 2007; Neish, 2009; Belkaid and Hand, 2014; Warner et al., 2016). However, the relationships between intestinal microbiota and IP have not yet been evaluated in a high-risk preterm population. In this study, we particularly focus on the intestinal immaturity in the challenging, yet most demanding population of very early gestational age subjects, and investigated the early development of the intestinal microbiota and its association with IP in preterm infants during the first 2 weeks of life. We observed that neonatal factors known to be associated with low IP, including early exclusive breast milk feeding and shorter duration of antibiotic exposure, significantly associate with the early colonization of the intestinal microbiota by members of Clostridiales. Given that Clostridiales and *Bifidobacterium* are most transcriptionally active and are also the most abundant bacterial groups in later ages (6–18 months), suggesting a process in which colonization with these two bacterial groups early during intestinal development after birth appears critical. The associations between neonatal factors, intestinal microbiota and intestinal barrier maturation further substantiate the multifactorial processes involved in intestinal barrier functions, thus highlighting the impact of neonatal care practices and the potential for therapies such as rationally designed live biotherapeutics strategies to rapidly lower IP after birth in preterm infants.

A critical value of understanding the driver of IP, including associated microbiological biomarkers, is in its clinical significance in NEC risk diagnostics. Further, it will also help identify the “window of opportunity” for strategizing intervention on leaky gut prior to the onset of NEC in disease prevention. Aberrant intestinal barrier function manifests by persistently high and/or late decrease in IP and is likely due to the physiological immaturity of the GI tract barrier function and altered levels of the normal microbial communities (Fitzgibbons et al., 2009; Fox and Godavitarne, 2012), resulting in microbial invasion of the intestinal wall and gut lamina propria triggering a cascading inflammatory response and ultimately intestinal necrosis and severe infection in NEC (Fasano, 2008; Mai et al., 2011; Nanthakumar et al., 2011; Neu and Walker, 2011; Bergmann et al., 2013). Multiple studies have revealed microbial community dysbiosis, characterized by the presence of members of the family *Enterobacteriaceae*, is involved in stimulating a hyperinflammatory response that leads to NEC (Mai et al., 2011; Nanthakumar et al., 2011; Neu and Walker, 2011; Bergmann et al., 2013; Carlisle and Morowitz, 2013; Ward et al., 2016). However, a generalized bacterial dysbiosis alone does not adequately explain NEC. Many preterm infants that are colonized by high abundance of *Enterobacteriaceae* do not develop NEC (La Rosa et al., 2014), and many NEC cases lacked intestinal colonization of *Enterobacteriaceae* (Barron et al., 2017). These results emphasize the importance of a holistic understanding of the etiology of NEC, including the mechanistic characterization of epithelial barrier mucosal immune response and microbiological stimulant, nutritional factors, *etc*. Early NEC risk assessment and prevention will ultimately improve overall neonate survival rates.

Multiple intrinsic and extrinsic factors affect newborn intestinal microbiota, such as maternal diet, delivery mode, breast milk feeding, antibiotic exposure, and other early life environmental exposures (Penders et al., 2006; Madan et al., 2012). Early exclusive breast milk feeding and shorter duration antibiotic exposure was observed to be associated with improved IP as well as increased abundance of members of Clostridiales in preterm infants (Saleem et al., 2017). Interestingly, these two neonatal factors have been shown to be critically protective against NEC (Colaizy et al., 2016). Clostridiales strains are recently shown to be susceptible to many antibiotics including ampicillin (Narushima et al., 2014), an antibiotic that is commonly used in clinic management in the first week of life. Our study showed Clostridiales was the most abundant taxonomic groups in old age group (6-18 mo), and it is in low abundance but high prevalence in the first 2 weeks of life, highlighting the importance of interventions such as limiting exposure to antibiotic in the NICU and development of a nutritional supplement for clinical administration for preterm infants. Further understanding of the selective nutritional requirement that favor the growth of the desired bacteria would afford the development of novel nutritional supplemental strategies to improve intestinal maturation and improve clinical outcomes in preterm infants.

The demonstrated association between IP, intestinal microbiota, and neonatal factors in this study suggest Clostridiales offer a new opportunity to develop a LBP for the early prevention of NEC, possibly in combination with strains of *Lactobacillus* and *Bifidobacterium* already available. Live biotherapeutics products (LBP) therapies are promising, low-cost, and constitute a likely safe preventive measure to improve intestinal barrier maturation and reduce NEC incidence in at-risk preterm infants (Stratiki et al., 2007). There have been at least 11 randomized controlled trials and a recent meta-analysis of LBP supplementation to prevent NEC in preterm neonates (Deshpande et al., 2010; Bergmann et al., 2013). Although there was a 30% reduction in NEC incidence in these trials, various formulations, doses, and duration of therapy were used, infants < 1000 g BW with the highest NEC incidence were under-represented, and no Food and Drug Administration-approved products are available to assure quality and safety under good manufacturing practices.

The taxonomic group Clostridiales was unfortunately known for a few pathogenic species that include *C. botulinum, C. perfringens, C. tetani*, and *C. difficile* in the family of *Clostridiaceae* (Rajilic-Stojanovic et al., 2007). However, Clostridiales has been largely overlooked because of the difficulties to culture *in vitro*. Recent application of culture-independent high-throughput sequencing identified many formerly unculturable Clostridiales species, and the group is now thought to be one of the predominant groups inhabiting the GI tract, comprising ∼30–40% abundance of the adult intestinal microbiota and 13 bacterial families, and majority are commensal of the intestinal microbiota (Walker et al., 2011). In fact, Clostridiales are heterogeneous in terms of their enzymatic and metabolic properties with anti-inflammatory properties, which often associates with their fermentative metabolism of carbohydrates and amino acids that produces beneficial short-chain fatty acid (SCFA) such as acetate, propionate, and butyrate (Smith et al., 2013; Stefka et al., 2014). Further, Clostridiales have been recently shown to stimulate the production of intestinal epithelial cytokines that have been associated with the improvement of intestinal dysbiosis, and marked reduction in inflammation (Atarashi et al., 2011, 2013; Narushima et al., 2014). The recent characterization of 46 strains of newly isolated Clostridiales revealed their ability to induce regulatory T cells and a protection against colitis and allergic responses (Atarashi et al., 2011). Seventeen strains of human-derived Clostridiales species were rationally selected using gnotobiotic mice and the cocktail shown to have prophylactic effect in mouse colitis (Atarashi et al., 2013; Narushima et al., 2014). In addition, the administration of Clostridiales protects the host from pathogen infection and abrogated intestinal pathology (McMurtry et al., 2015). In term infants, the presence of Clostridiales in the intestinal microbiota was demonstrated to prevent colonization by bacterial pathogens such as *S. Typhimurium* (Kim et al., 2017). These species form the basis of the microbiome therapeutics product, SER109, for the treatment of *C. difficile* infection in adults (Khanna et al., 2016). Future characterization of Clostridiales species will potentially prevent microbial community-mediated intestinal dysbiosis in preterm infants to optimize intestinal maturation and limit the burden of prematurity (Ward et al., 2016).

The current standard application of 16S rRNA V4 or V3-V4 amplicon sequencing is unfortunately not capable to resolve the species of Clostridiales present in a sample (Jovel et al., 2016). The genome referencing database of Clostridiales has been rapidly increasing while still is largely biased toward pathogenic strains, and many Clostridiales commensal strains still remain unclassified (NCBI, 2018). Future bacterial cultivation and genome characterization of targeted members of Clostridiales will greatly improve our capability to develop novel diagnostic and treatment strategies for intestinal barrier immaturity. We also acknowledge the limitations and challenges in longitudinally collected fecal samples from preterm infants at early gestation (<33 weeks) in NICU, when regular bowel movement has not yet been established so that certain time points, particularly the first event of meconium poop, can be failed to catch within the collection timeframe. Future studies on an expanded cohort size will further help to amend the missing data issue and enable large-scale statistical modeling of longitudinally microbiota change in preterm infants. Another main limitation is that the association between intestinal permeability and the microbiological biomarker defined in this study, albeit highly significant, could not establish the mechanistic role and cause-effect relevance governing intestinal barrier maturation and the microbial biomarkers. The associations observed in this study among neonatal factors, intestinal microbiota, and intestinal barrier maturation further substantiate the multifactorial processes involved in intestinal barrier functions. Future animal model and/or organoid studies could further assist mechanistic understanding of intestinal barrier maturation during the first few days of life for future assessment of their therapeutic value in clinical practice.

## Materials and Methods

### Participants and Intestinal Permeability Measurement

This study was approved and carried out in accordance with the recommended protocol by the institutional review boards of the University of Maryland and Mercy Medical Center. All subjects have obtained written informed parental consent in accordance with the approved protocol. All methods were performed in accordance with the relevant guidelines and regulations. Thirty-eight preterm infants 24^0/7^–32^6/7^ weeks GA were enrolled within 4 days after birth and received 1 ml/kg of the non-metabolized sugar probes lactulose (La) (marker of intestinal paracellular transport)/rhamnose (Rh) (marker of intestinal transcellular transport) (8.6 g La +140 mg Rh/100 mL) enterally within 24 h of enrollment on study day 1, and subsequently on study day 8 ± 2 and 15 ± 2. La/Rh was measured by high-pressure liquid chromatography (HPLC) in urine collected over a 4 h period following administration of the sugar probes as previously described (Saleem et al., 2017). High or low intestinal permeability was defined by a La/Rh > 0.05 or ≤0.05 respectively, as validated and applied previously (Saleem et al., 2017). PMA at each study timepoint was calculated as gestational age at birth plus postnatal age as defined previously (Grier et al., 2017). Fecal samples (∼1 g) were collected at the same time as the dual sugar administration and stored immediately in 2 ml of RNAlater (QIAGEN). Urine and fecal samples were archived at -80°C until processed. A standard feeding protocol was used for all preterm participants. To compare microbiota of infants at different growth phases (Mshvildadze et al., 2008; Unger et al., 2015), 16 samples from older term infants at phase II/III (6–24 months old) from a previous study (Sellitto et al., 2012) were included in the comparative analyses.

### Stool Nucleic Acid Extraction and Sequencing

DNA was extracted from all samples as previously reported (Ravel et al., 2011). Briefly, a 500 μl aliquot of fecal material mixture was homogenized and carefully washed twice in PBS buffer. Enzymatic lysis using mutanolysin, lysostaphin and lysozyme was performed, followed by proteinase K, SDS treatment and bead beating. DNA purification from lysates was done on a QIAsymphony automated platform. PCR amplification of the V3–V4 variable region of 16S rRNA gene was performed using dual-barcoded universal primers 319F and 806R as previously described (Fadrosh et al., 2014). High-throughput sequencing of the amplicons was performed on an Illumina MiSeq platform using the 300 bp paired-end protocol. Metagenomic sequencing libraries were constructed from the same DNA using Illumina Nextera XT kit according to the manufacturer recommendations.

Stool specimen was advised to be collected within the stool mass as much as feasible from diaper to avoid frequent air exposure. The stool sitting time is 0–3 h and was collected during diaper change every 3 h in NICU. Stool specimen were stored immediately in RNALater that stabilizes and protects the integrity of RNA to minimize the need to immediately process or freeze specimen (Gorokhova, 2005). The use of metatranscriptome in microbiome study is still a very young field, intensive evaluations such as RNA consistency over time in stool diaper collection are needed for future validation. Total RNA was extracted from 250 μl of stool homogenized in RNALater. Briefly, lysis was performed by bead beating using the FastPrep lysing matrix B protocol (MP Biomedicals), followed with two rounds of protein cleanup using phenol-chloroform in 5PRIME heavy phase lock tubes (QuantaBio) and precipitation of total nucleic acids using isopropanol. Genomic DNA was removed using TURBO DNase (Ambion). Ribosomal RNAs were depleted using the Gram-negative and Human/mouse/rat Ribo-Zero rRNA Removal kits (Epicentre Technologies). The resulting RNA was used for library construction using Illumina TruSeq stranded mRNA library preparation kit according to the manufacturer’s recommendations. Quantification of the constructed RNA libraries was performed on an Agilent Bioanalyzer using the DNA 1000 Nano kit. Both metagenome and metatranscriptome samples were sequenced on an Illumina HiSeq 4000 instrument at the Genomics Resource Center (GRC), Institute for Genome Sciences, University of Maryland School of Medicine using the 150 bp paired-end protocol.

### Bioinformatics Analysis of Intestinal Microbiota

Sequencing read quality assessment was performed using strict criteria to ensure high quality and complete sequences of the amplified the V3–V4 regions of the 16S rRNA gene, according to the procedures, programs and citations, and parameters described previously (Fadrosh et al., 2014). Briefly, a sequence read was trimmed at the beginning of a 4 bp sliding window if the average quality score was less than Q15. The sequence read was then assessed for length and retained if it was at least 75% of its original length. The paired-end reads were assembled to take advantage of the ∼90 bp overlapping region. These sequences were further de-multiplexed the sequence reads by individual samples. Additional quality filtering was applied that removed sequences with more than one mismatch in the barcode sequence tag or with ambiguous nucleotide. Taxonomic assignments were performed on each sequence using the Ribosomal Database Project trained on the Greengene database (Aug 2013 version), using 0.8 confidence values as cutoff. Clustering taxonomic profiles was performed as previously described (Ravel et al., 2011). The number of clusters was validated using gap statistics implemented in the *cluster* package in R (Maechler, 2016) by calculating the goodness of clustering measure. Within-sample diversity was estimated using both observed OTUs to measure community richness and Shannon diversity index. Linear discriminant analysis (LDA) effect size (LEfSe) analysis (Segata et al., 2011) was used to identify fecal phylotypes that could explain the differences between infants with low or high La/Rh ratio on different sampling days. For LEfSe, the alpha value for the non-parametric factorial Kruskal-Wallis (KW) sum-rank test was set at 0.05 and the threshold for the logarithmic LDA model (Fisher, 1936) score for discriminative features was set at 2.0. An all-against-all BLAST search in multi-class analysis was performed. Balance tree analysis was applied as implemented in Gneiss, and trees were generated using Ward hierarchical clustering of abundance profiles. Balance was computed as the isometric log ratio of mean abundances at each bifurcating node in the tree, to characterize the “flow” of changes in the abundance of a group of correlated bacteria in a microbial community (Morton et al., 2017). Multivariate response linear regression on the calculated balances was performed, and multiple factors were included as covariates, including antibiotics use, maternal antibiotics use, delivery mode, preterm premature rupture of membranes, feeding pattern and source, intestinal permeability, birthweight, gender, ethnicity, GA and PMA. Leave-one-variable-out approach was used to calculate the change in R square to evaluate the effect of a single covariate on the community. Ten-fold cross validation was performed to mitigate the common overfitting issues in statistical modeling.

### Statistical Analysis

An adaptive spline logistic regression model implemented in spmrf R package (Faulkner and Minin, 2018) was adapted to determine the associations between intestinal permeability and relative abundance of bacterial phylotypes. This model is a locally adaptive non-parametric fitting method that operates within a Bayesian framework, which uses shrinkage prior Markov random fields to induce sparsity and provides a combination of local adaptation and global control (Faulkner and Minin, 2018). The analysis was performed on the phylotypes present in at least 15% of all samples, and the effect size was defined as the difference between the extreme values of the probability of intestinal permeability index. Given that there were multiple samples collected from each subject, this model takes into consideration of the dependencies among samples within a subject. Bayesian goodness-of-fit *p*-value implemented in R package rstan (Team, 2018) was used to access the significance of the association between phylotypes and metadata including antibiotics use, maternal antibiotics use, delivery mode, PPROM, feeding pattern, intestinal permeability, birthweight, gender, ethnicity, gestational age, and postmenstrual age. R code implementation of the model is provided in Supplementary File S1. We further adapted random forest supervised machine learning scheme implemented in R package randomForest (Liaw and Wiener, 2002) to test the predictability of the phylotypes of microbial community on intestinal permeability. The top 15 phylotypes relative abundance with highest mean decrease gini index importance measure, were fitted to a random effect logistic regression model of intestinal permeability that was defined as a dichotomous variable high (La/Rh > 0.05) or low (La/Rh ≤ 0.05). The relative abundances of phylotypes were centered to the mean and scaled by standard deviation to apply to the model to normalize relative abundances. R code implementation of the model is provided in Supplementary File S2.

### Intestinal Microbiome Analyses

Metagenomic and metatranscriptomic sequence data were pre-processed using the following steps: (1) human sequence reads and rRNA LSU/SSU reads were removed using BMTagger v3.101 (Rotmistrovsky and Agarwala, 2011) using a standard human genome reference (GRCh37.p5) (Church et al., 2011); (2) rRNA sequence reads were removed *in silico* by aligning all reads using Bowtie v1 (Langmead et al., 2009) to the SILVA PARC ribosomal-subunit sequence database (Quast et al., 2013). Sequence read pairs were removed even if only one of the reads matched to the human genome reference or to rRNA; (3) the Illumina adapter was trimmed using Trimmomatic (Bolger et al., 2014); (4) sequence reads with average quality greater than Q15 over a sliding window of 4 bp were trimmed before the window, assessed for length and removed if less than 75% of the original length; and (5) no ambiguous base pairs were allowed. The taxonomic composition of the microbiomes was established using MetaPhlAn version 2 (Segata et al., 2012). Normalization using Witten-Bell smoothing was performed since metatranscriptomes are a random sampling of all expressed genes and transcripts can be identified that correspond to genes not represented in the metagenome, particularly for low abundance species that were metabolically active (Franzosa et al., 2014). The relative expression of a gene in a sample was calculated by normalizing the smoothed value of the expression level in the metatranscriptome by the smoothed value of the corresponding gene abundance in the metagenome, as suggested previously (Franzosa et al., 2014, 2015). Correlation plots were generated using R *corrplot* package (Wei and Simko, 2017). Genotypic variation of *Escherichia coli* was performed through reconstructing MLST loci-sequences from metagenomes using metaMLST program (Zolfo et al., 2017). The resulting STs were visualized to show related genotypes of *E. coli* strains on a minimum spanning tree computed by a goeBURST algorithm (Francisco et al., 2009) implemented in PHYLOViZ (Nascimento et al., 2017).

## Conclusion

At birth there is low abundance of Clostridiales in preterm infants with progressive, significant increase in abundance in the group with rapid progression toward intestinal barrier maturation, but remained low in those with persistent high IP over the first 2 weeks of life. We further observed neonatal factors previously identified to promote intestinal barrier maturation, including early exclusive breastmilk feeding and shorter duration antibiotic exposure that are associated with the early colonization of the intestinal microbiota by members of the Clostridiales, which altogether are associated with improved intestinal barrier function in preterm infants. This highlights the importance of factors such as breast milk feeding and limiting exposure to antibiotic in the high-risk preterm population. Our study suggests Clostridiales can potentially be developed as a rapid, non-invasive screening tool of leaky gut and early prevention of NEC to identify “at-risk” preterm infants. Further, our study suggests rationally selected and formulated Clostridiales species could constitute a promising LBP candidate to foster intestinal barrier function maturation and potentially reduce risk for NEC, especially when combined with already available strains of *Bifidobacterium* and *Lactobacillus*. The rationale for this intervention is supported by our correlative finding between increased Clostridiales abundance and intestinal barrier maturation of preterm neonates at-risk for NEC development. Identification of specific strains of Clostridiales, their functions in mediating intestinal barrier maturation, LBP formulation and manufacturing, dosing, safety and efficacy evaluation will be needed to support their application as oral supplementation to promote intestinal barrier maturation and overall health of preterm neonates. Early prediction and prevention of NEC will ultimately improve overall infant survival rates.

## Data Availability

All 16S rRNA sequence data were deposited in SRA SRP132205 (https://www.ncbi.nlm.nih.gov/Traces/study/?acc=PRJNA432222) under BioProject PRJNA432222 (https://trace.ncbi.nlm.nih.gov/Traces/sra/sra.cgi?study=SRP132205). Metatranscriptome data were deposited in European Nucleotide Archive PRJEB29365 for open assessment.

## Author Contributions

BM, AO-W, AF, JR, and RV designed the research. AO-W and RV conducted the clinical study. BM, EM, HY, and MH performed the microbiota analyses. BM and PG performed the statistical analyses. BM, EM, JR, and RV wrote the paper.

## Conflict of Interest Statement

The authors declare that the research was conducted in the absence of any commercial or financial relationships that could be construed as a potential conflict of interest.

## Supplementary Material

FIGURE S1 Changes of intestinal permeability of each subject at study day 1, 8, and 15. Circle dot represents a sampling point, the line collecting points represents each subject at different time point. Different color of the lines specifies different subjects. The low and high intestinal permeability category was defined by a La/Rh > 0.05 or ≤0.05 respectively.

FIGURE S2 Heatmap of the 50 most abundant intestinal bacterial taxa relative abundance in samples collected from 38 preterm infants enrolled in the study. The microbiota of 64 fecal samples were successfully characterized by high-throughput sequencing of the V3–V4 variable regions of 16S rRNA genes. The three sidebars indicate cluster, time, and intestinal permeability category, respectively. Ward linkage clustering was used to cluster samples based on their Jensen-Shannon distance calculated in vegan package in R (Oksanen et al., 2011). The samples with no IP assessment were included to generated the clusters. The low and high intestinal permeability category was defined by a La/Rh > 0.05 or ≤0.05 respectively (Saleem et al., 2017). Taxonomic profiling of corresponding metagenomes further resolved *Klebsiella* spp. to *Klebsiella pneumoniae, Enterococcus* spp. to *Enterococcus faecalis*, and *Bifidobacterium* spp. to *Bifidobacterium breve*.

FIGURE S3 Prediction and residual plots of balance tree analysis. **(A)** The projected residuals of input and prediction onto the plotted balances. The predicted points lie within the same region as the original communities, indicating a high credibility of the prediction. **(B)** The residuals have roughly the same variance as the predictions and are mostly within ±2. The top two balances are plotted, and the percentage of variance described by the plotted balances is indicated on the axes.

FIGURE S4 Comparison of the relative abundance of members of *Clostridiales* **(A)**, *Bifidobacteriales* **(B)**, *Staphylococcaceae* **(C)**, and *Enterobacteriaceae* **(D)** on phase I (1–15 days old) and III (6–18 months old) infants (Unger et al., 2015). Bars represent the relative abundance of each bacterial group in each sample. Dotted line represents mean, solid line represents median relative abundance. The alpha value for the non-parametric factorial Kruskal-Wallis sum-rank test was 0.05 and the threshold for the logarithmic LDA model (Fisher, 1936) score for discriminative features was set at 2.0.

FIGURE S5 Dependence between intestinal permeability binary index and the log relative abundance of *Coprococcus* **(A)** and *Clostridiales* **(B)**. An adaptive spline logistic regression model implemented in spmrf R package was applied to the phylotypes present in at least 15% of all samples. Low IP: La/Rh < 0.05; high IP: La/Rh ≥ 0.05. Bar on x-axis indicates the dichotomous categories in intestinal permeability: gray: low IP; red: high IP.

FIGURE S6 Mean decrease Gini index random forest importance measure for the classification of intestinal permeability using relative abundance of bacterial phylotypes. Top 30 measurements were shown on the figure, and the top 15 phylotypes were used to fit a random effect logistic regression model of IP. Measurement is calculated using random forest supervised machine learning scheme implemented in R package randomForest (Liaw and Wiener, 2002). Low IP: La/Rh < 0.05; high IP: La/Rh ≥ 0.05.

FIGURE S7 Correlation plot between the bacterial species that are most correlated in normalized transcriptome profiles. Correlation plots were generated using *corrplot* package (Wei and Simko, 2017) in R. Spearman correlation coefficients are shown in figure. *Y* and *x*-axis indicate the relative abundance on logarithm 10 scale of the bacterium labeled.

FIGURE S8 Dependence between time reaching full breastmilk feeding and the log relative abundance of *Clostridiales* **(A)** and *Klebsiella* **(B)**. An adaptive spline logistic regression model implemented in spmrf R package was applied to the phylotypes present in at least 15% of all samples. More than 10 postnatal days to reach full breastmilk feeding is considered late time, while less than 10 postnatal days is considered early time. Bayesian goodness-of-fit *p*-value implemented in R package rstan (Team, 2018) was used to access the significance of the association between phylotypes and investigated factors. Bar on x-axis indicates the dichotomous categories in time point reaching full breast milk feeding: gray: earlier time (<10 days); red: late time (≥10 days).

FIGURE S9 Dependence between duration of antibiotic treatment and the log relative abundance of *Clostridiales* **(A)** and *Lachnospiraceae* **(B)**. An adaptive spline logistic regression model implemented in spmrf R package was applied to the phylotypes present in at least 15% of all samples. Use of antibiotics for more than 4 days was considered long duration, while less or equal to 4 days was considered short duration. Bayesian goodness-of-fit *p*-value implemented in R package rstan (Team, 2018) was used to access the significance of the association between phylotypes and investigated factors. Bar on *x*-axis indicates the dichotomous categories in antibiotic treatment duration: gray: shorter duration (<4 days); red: longer duration (≥4 days).

FILE S1 R markdown code implementation of the adaptive spline logistic regression model using the *spmrf* R package (Faulkner and Minin, 2018).

FILE S2 R markdown code implementation of the random forest supervised machine learning scheme using the R package randomForest (Liaw and Wiener, 2002).

FILE S3 Taxonomic table and metadata used in this study. **(A)** The demographic, obstetric, and neonatal characteristics for each subject included in this study and the phylotype using high-throughput sequencing of the V3-V4 variable regions of 16S rRNA genes. B Taxonomic profiles of stool samples from 16 infants at 6-24 months (Phase II/III) of age born at term, using high-throughput sequencing of the V3-V4 variable regions of 16S rRNA genes. **(C)** Taxonomic profiles of 19 stool samples, using metagenome sequencing and profiled using MetaPhlAn version 2 (Segata et al., 2012).

FILE S4 Statistics summary of Gneiss balance tree analysis using the simplicial linear regression (Morton et al., 2017). Covariates of antibiotics use, maternal antibiotics use, delivery mode, preterm premature rupture of membranes (pPROM), feeding pattern, IP, birthweight, gender, ethnicity, gestational age (GA) and postmenstrual age (PMA) were included in the analysis.

FILE S5 Statistics summary of random effect logistic regression model of intestinal permeability and associations with relative abundance of bacterial phylotypes. The top 15 phylotypes with the highest mean decrease gini index importance measure listed in Supplemental Figure 5 were included in this analysis.
