# Supplementary material for: Microbial Biomarkers of Intestinal Barrier Maturation in Preterm Infants
Source: Front Microbiol. 2018 Nov 14;9:2755. doi: 10.3389/fmicb.2018.02755 (PMC6246636; doi:10.3389/fmicb.2018.02755)
Supplement: FIGURE S1 — Changes of intestinal permeability of each subject at study day 1, 8, and 15. Circle dot represents a sampling point, the line collecting points represents each subject at different time point. Different color of the lines specifies different subjects. The low and high intestinal permeability category was defined by a La/Rh > 0.05 or ≤0.05 respectively. [file Data_Sheet_1.zip › Supplementary_information/Supplemental_file/Supplementary File S1.pdf]

# Dependence of IP and IPcat on phylotype relative abundances: binomial and continuous models utilizing spmrf adaptive splines

```
pacman::p_load(rstan,car)
load("../2nd/spmrf_normal_horseshoe_order2.rda")
load("../2nd/spmrf_bernoulli_horseshoe_order2.rda")
source("../2nd/stan_utils_bma.R") # from https://github.com/betanalpha/knitr_case_studie
s/tree/master/rstan_workflow
load("../1st/perm3.RData") # mt, ct, pt, bt, phProp, selPh.5 [1:166], selPh.10 [1:124],
 selPh.15 [1:88], selPh.20 [1:70], selPh.25 [1:55] #241 OTU and 31 meta
```

```
## plot spmrf spline of log10(IP) vs log10(rel abundance) of a specified phylotype
## and pr( IPcat ) vs log10( rel abundance) of a specified phylotype
plot.IP.spmrf <- function(ph, nItr=2000, thin=1, nChains=3, nCores=3, mar=c(4.0, 4.0, 0.
5, 0.5), mgp=c(2.5,0.6,0), alpha=0.05) {
  ## continues model
  p <- pt[,ph]
  x <- log10(p)
  y <- log10(mt$IP)
  idx <- is.finite(x) & is.finite(y)
  x <- x[idx]
  y <- y[idx]
  o <- order(x)
  x <- x[o]
  y <- y[o]
  length(x)

  m.dat <- list(y=y, xvar1=x, J=length(y))
  m.dat2 <- spmrf.get.data(m.dat)

  m1 <- sampling(spmrf.normal.o2.model, data=m.dat2, control=list(adapt_delta=0.999, max
_treedepth=12), iter=nItr, thin=thin, chains=nChains, cores=nCores)

  plow <- alpha/2
  phigh <- 1 - alpha/2

  theta <- rstan::extract(m1, "theta")[[1]]
  y.med <- apply(theta, 2, median)
  y.mad <- apply(theta, 2, mad)
  y.l <- apply(theta, 2, quantile, probs = plow)
  y.u <- apply(theta, 2, quantile, probs = phigh)

  r2 <- gEff.pval(x, y.med, y.mad)

  gEff.ip <- numeric(3)
  names(gEff.ip) <- c("gEff", "p-val","median($\\log_{10}$(RA))")
  gEff.ip <- c(r2$eff, r2$pval, median(x))

  x.uq <- unique(m.dat2$xvar1)
  ylim <- range(c(y.l, y.u, y))

  lmat <- matrix(c(1,1,2,3),nrow=2,byrow=T)
  lhei <- c(1, 10, 10)
  layout(lmat, heights = lhei, respect = F)

  ## title
  op <- par(mar=rep(0,4), mgp=mgp, tcl = -0.3)
  plot(1,1, axes=F, xlab="", ylab="", type='n')
  text(1, 1, labels=paste0(ph, "  (n=", length(x),")"), font=2, cex=1.5)
  par(op)

  op <- par(mar=mar, mgp=mgp, tcl = -0.3)
  plot(x.uq, y.med, type = "n", las=1, ylim=ylim, ylab="IP", xlab="log10( Relative Abund
ance )")
```

```
  polygon(c(x.uq, rev(x.uq)), c(y.l, rev(y.u)),  border = NA, col='gray90')
  lines(x.uq, y.med, col="blue", lwd=1)
  points(x,y)
  par(op)

  ## binary model
  p <- pt[,ph]
  x <- log10(p)
  y <- ifelse(mt$IP_category=="low",0,1)
  idx <- is.finite(x) & is.finite(y)
  x <- x[idx]
  y <- y[idx]
  o <- order(x)
  x <- x[o]
  y <- y[o]
  length(x)

  m.dat <- list(y=y, xvar1=x, J=length(y))
  m.dat2 <- spmrf.get.data(m.dat)

  m2 <- sampling(spmrf.bernoulli.o2.model, data=m.dat2, control=list(adapt_delta=0.999,
 max_treedepth=12), iter=nItr, thin=thin, chains=nChains, cores=nCores)

  theta <- rstan::extract(m2, "theta")[[1]]
  y.med <- apply(theta, 2, median)
  y.mad <- apply(theta, 2, mad)
  y.l <- apply(theta, 2, quantile, probs = plow)
  y.u <- apply(theta, 2, quantile, probs = phigh)

  r2 <- gEff.pval(x, y.med, y.mad)

  gEff.ip.cat <- numeric(3)
  names(gEff.ip.cat) <- c("gEff", "p-val","median($\\log_{10}$(RA))")
  gEff.ip.cat <- c(r2$eff, r2$pval, median(x))

  x.uq <- unique(m.dat2$xvar1)
  ylim <- range(c(y.l, y.u))

  op <- par(mar=mar, mgp=mgp, tcl = -0.3)
  plot.logit(x.uq, y[seq(x.uq)], expit(y.med), expit(y.l), expit(y.u), title="", ylab="p
r( IP category )", xlab="log10( Relative Abundance )")
  par(op)

  list(gEff.ip=gEff.ip, gEff.ip.cat=gEff.ip.cat, ph=ph)
}
```

```
res15 <- list()
for ( i in seq(selPh.15) ) {
    ph <- selPh.15[i]
    file <- paste0("./pics/",i,"_spmrf_plot_selPh15_v3.pdf")
    pdf(file, width=12, height=6)
    op <- par(mar=c(4.0, 4.0, 0.5, 0.5), mgp=c(2.5,0.4,0),tcl = -0.3)
    res15[[i]] <- plot.IP.spmrf(ph)
    par(op)
    dev.off()
}

gEff.IP.15 <- matrix(nrow=length(selPh.15), ncol=4)
colnames(gEff.IP.15) <- c("gEff", "p-val","q-val","median($\\log_{10}$(RA))")
rownames(gEff.IP.15) <- selPh.15

gEff.IPcat.15 <- matrix(nrow=length(selPh.15), ncol=4)
colnames(gEff.IPcat.15) <- c("gEff", "p-val","q-val","median($\\log_{10}$(RA))")
rownames(gEff.IPcat.15) <- selPh.15

for ( i in seq(selPh.15) ) {
    ph <- selPh.15[i]
    r <- res15[[i]]
    gEff.IP.15[ph, c(1:2,4)] <- r$gEff.ip
    gEff.IPcat.15[ph, c(1:2,4)] <- r$gEff.ip.cat
}

gEff.IP.15[,3] <- p.adjust(gEff.IP.15[,2], method="fdr")
gEff.IPcat.15[,3] <- p.adjust(gEff.IPcat.15[,2], method="fdr")

o <- order(gEff.IP.15[,2])
gEff.IP.15 <- gEff.IP.15[o,]

o <- order(gEff.IPcat.15[,2])
gEff.IPcat.15 <- gEff.IPcat.15[o,]
```

```
##                                                               gEff
## k__Bacteria.p__Firmicutes.c__Clostridia.o__Clostridiales -0.6012381
## k__Bacteria.p__Firmicutes.c__Clostridia                  -0.5984238
##                                                              p-val
## k__Bacteria.p__Firmicutes.c__Clostridia.o__Clostridiales 0.01489097
## k__Bacteria.p__Firmicutes.c__Clostridia                  0.01746812
##                                                            q-val
## k__Bacteria.p__Firmicutes.c__Clostridia.o__Clostridiales 0.492801
## k__Bacteria.p__Firmicutes.c__Clostridia                  0.492801
```

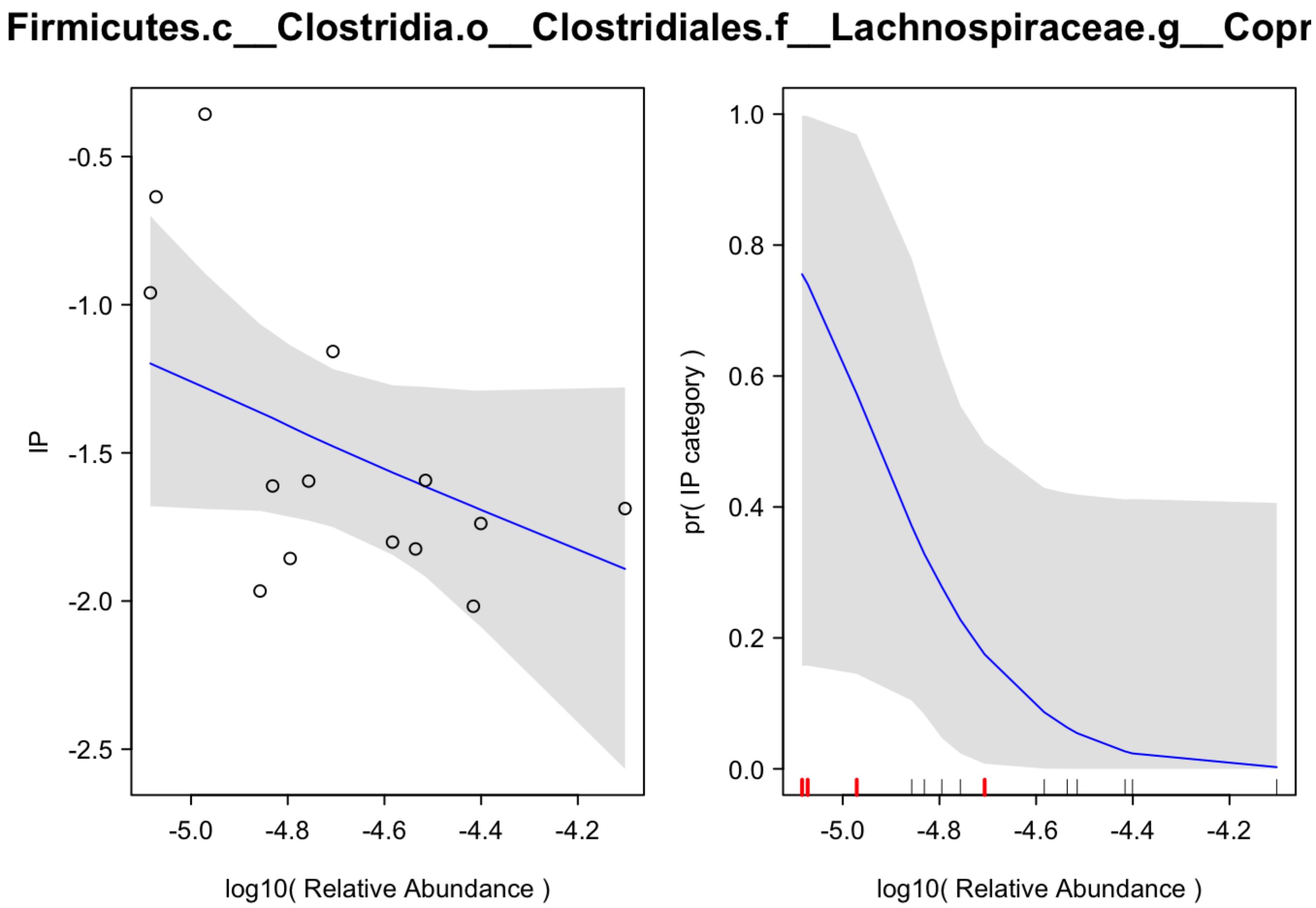


Plot1

```
## $gEff.ip
## [1] -0.10071488  0.02664069 -4.73165485
##
## $gEff.ip.cat
## [1] -0.75282970  0.09211407 -4.73165485
##
## $ph
## [1] "k__Bacteria.p__Firmicutes.c__Clostridia.o__Clostridiales.f__Lachnospiraceae.g__C
oprococcus"
```

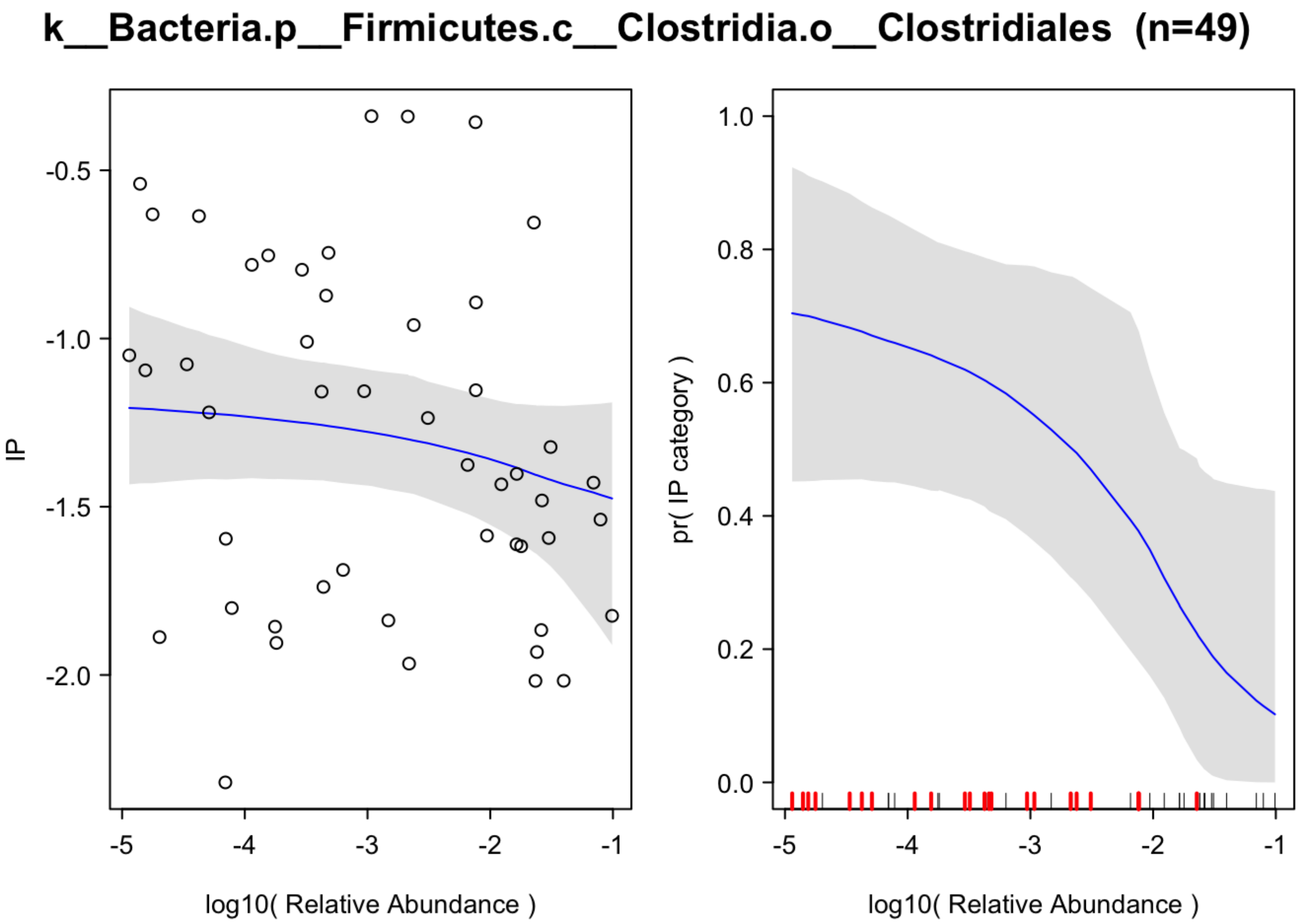


Plot2

```
## $gEff.ip
## [1] -0.04423125  0.07080473 -2.83014603
##
## $gEff.ip.cat
## [1] -0.60193284  0.01192112 -2.83014603
##
## $ph
## [1] "k__Bacteria.p__Firmicutes.c__Clostridia.o__Clostridiales"
```
