# Supplementary material for: Microbial Biomarkers of Intestinal Barrier Maturation in Preterm Infants
Source: Front Microbiol. 2018 Nov 14;9:2755. doi: 10.3389/fmicb.2018.02755 (PMC6246636; doi:10.3389/fmicb.2018.02755)
Supplement: FIGURE S1 — Changes of intestinal permeability of each subject at study day 1, 8, and 15. Circle dot represents a sampling point, the line collecting points represents each subject at different time point. Different color of the lines specifies different subjects. The low and high intestinal permeability category was defined by a La/Rh > 0.05 or ≤0.05 respectively. [file Data_Sheet_1.zip › Supplementary_information/Supplemental_file/Supplementary File S2.pdf]

16S rRNA analysis of permeability data

```
pacman::p_load(lme4,rjags)
load("../3rd/IP_16S_OTUs_w_metadata.RData") # tt, ct, pt

idx <- !is.na(tt$Lac_Rh_ratio)
ct <- ct[idx,]
pt <- pt[idx,]

subjID <- tt[rownames(ct), "subjectID"]
names(subjID) <- rownames(ct)
```

```
pacman::p_load(randomForest)

## version of rf.cv() with cross-validation done at the subject level
rf.cv2 <- function(X, y, subjID, nfolds=10, verbose=FALSE, ... ) {
    ## runs
    ## k-fold cross-validation of randomForest on (X,y)
    ## returns

    ## Arguments:
    ## X      - predictors; data frame or matrix
    ## y      - response; vector of the samle length as nrow(X)
    ## subjID - vector of length nrow(X) with subject assignment to each sample
    ## nfolds - number of folds
    ## verbose - if print progress message
    ## ... - parameters to pass to randomForest()

    ## Values:
    ## - error:  list of differences between prediction and true values for
    ##   regression and pred==true logical vectors for classification (one for each
    ##   fold)

    ## - rmse: root mean squared error

    ## - nmse: normalized MSE = MSE/MSE(mean(y) as predictor) for regression and
    ##   MSE/MSE(the highest frequency class as predictor ) for classification

    ## - mae: mean absolute error)

    ## - nmae: normalized MAE

    ## - cl.err: classification error = accuracy = sum(pred!=y)/length(y)

    n <- length(y)

    if ( !is.numeric(y) && !is.factor(y) )
        stop("y should be either numeric or factor")

    if ( !is.data.frame(X) && !is.matrix(X) )
        stop("X is neither data frame nor matrix")

    if ( n!= nrow(X) )
        stop("n!= nrow(X)")

    if ( n!= length(subjID) )
        stop("n!= length(subjID)")

    if ( !is.numeric(nfolds) )
        stop("nfolds is not numeric")

    if ( nfolds < 1 )
        stop("nfolds < 1")

    if ( nfolds > n )
```

```
        stop("nfolds > n")

    if ( length(y[is.na(y)]) > 0 ) {
        warning("y has some NAs; removing them and the corresponding rows of X and subjI
D")
        idx <- !is.na(y)
        y <- y[idx]
        X <- X[idx,]
        subjID <- subjID[idx]
    }

    subjID <- as.character(subjID)
    uqSubjIDs <- unique(subjID)
    nSubj <- length(uqSubjIDs)

    if ( nfolds > nSubj ) {
        warning(paste("nfolds needs to be not greater than number of subjects. Changing
 it to number of subjects: ",nSubj, sep=""))
        nfolds <- nSubj;
    }

    sp <- split(1:n, list(factor(subjID))) # list with each entry being a
                                           # vector of indices of y that
                                           # correspond to the same subjID
                                           # (used as the label of the list
                                           # element)

    y.mostFr <- NA
    if ( is.numeric(y) ) {
        ##
    } else {
        ## splitting should preserve the frequency of factors
        lev <- levels(y)
        fqTbl <- table(y)
        y.mostFr <- names(fqTbl)[which.max(fqTbl)]
    }

    ##     # The splitting of data is done on the subject level. In particular, if
    ##     # nfolds is equal to the number of subjects, we get a jack-knif
    ##     # leave-one-out CV.
    ##     # Note that y does not have to be constant over subjects.

    s0 <- split(sample(nSubj),rep(1:nfolds,length=nSubj)) # nfolds split of all subjects
    ## Turning each element of s0 from vector of subject indices to vector of
    ## sample indices corresponding to the given subjects
    s <- list()
    for ( i in seq(s0) ) {
        v <- uqSubjIDs[s0[[i]]]
        s[[i]] <- as.vector(unlist(sp[v]))
    }
    x.null <- rep(y.mostFr, n)

    error <- list()      # list of prediction errors: prediction - y
    error.null <- list() # list of null model prediction errors: mean(y) - y;
```

```
                          # mean(y) is predicted value for each coordinate - null
                          # model; I am returning error.null so we can test if the
                          # current model is significantly better than the null
                          # model - that is if the mean(abs(errors)) is
                          # significantly different from the
                          # mean(abs(null.errors))

    sampleIdx <- c()      # vector of sample indices from each run of cross validation, s
o we can match errors with samples
    r2.loc <- numeric(nfolds)
    r2.pearson.loc <- numeric(nfolds)
    r2.spearman.loc <- numeric(nfolds)
    rmse.loc <- numeric(nfolds)
    nmse.loc <- numeric(nfolds)
    mae.loc <- numeric(nfolds)
    nmae.loc <- numeric(nfolds)
    cl.err.loc <- numeric(nfolds)
    ncl.err.loc <- numeric(nfolds) # normalized classification error
    gError <- numeric(n) # "global" error array whose i-th entry is 1 is in the 10 fold
 CV the prediction of y[i] was correct
    imp <- matrix(0, nrow=ncol(X), ncol=2)
    y.pred.list <- list()
    for ( i in seq(nfolds) ) {
        if ( verbose )
            print(paste(" i=",i, sep=""))

        sampleIdx <- c(sampleIdx, s[[i]])

        trIdx <- setdiff(1:n, s[[i]])
        m.rf <- randomForest( X[trIdx,], y[trIdx], importance=TRUE, ... )
        y.pred <- predict(m.rf, newdata=X[s[[i]],], type="response")
        y.pred.list[[i]] <- y.pred

        if ( is.numeric(y) ) {
            y.mean <- mean(y[trIdx])
            error[[i]] <- y.pred - y[s[[i]]]
            error.null[[i]] <- y.mean - y[s[[i]]]
            rmse.loc[i] <- sqrt(mean( error[[i]]^2 ))
            r2.loc[i] <- 100 * ( 1 - sum( error[[i]]^2 ) / sum( (y.pred - y.mean)^2 ) )
# percentage of variance explained
            r2.pearson.loc[i] <- 100*cor(y.pred, y[s[[i]]])^2
            r2.spearman.loc[i] <- 100*cor(y.pred, y[s[[i]]], method="spearman")^2
            nmse.loc[i] <- mean( error[[i]]^2 ) / mean( (y.pred - y.mean)^2 )
            mae.loc[i] <- mean( abs( error[[i]] ) )
            nmae.loc[i] <- mean( abs( error[[i]] ) ) / mean( abs(y.pred - y.mean) )
            imp <- imp + m.rf$importance
        } else {
            m <- length(s[[i]])
            error[[i]] <- as.character(y.pred) != as.character(y[s[[i]]])
            error.null[[i]] <- as.character(x.null[1:m]) != as.character(y[s[[i]]])
            cl.err.loc[i] <- sum(error[[i]]) / m
            ncl.err.loc[i] <- cl.err.loc[i] / ( sum(error.null[[i]]) / m ) # NOTE that t
his will be NaN when the denominator is 0 (null model has no errors for the given y[s
[[i]]]
```

```
            imp <- imp + m.rf$importance[,3:4]
        }
        gError[s[[i]]] <- as.integer(error[[i]])
    }

    list(cv.list=s,
         y.pred.list=y.pred.list,
         error=error,
         error.null=error.null,
         sampleIdx=sampleIdx,
         imp=imp/nfolds,
         rmse=mean(rmse.loc), nmse=mean(nmse.loc),
         mae=mean(mae.loc), nmae=mean(nmae.loc),
         r2=mean(r2.loc),
         r2.pearson=mean(r2.pearson.loc),
         r2.spearman=mean(r2.spearman.loc),
         gError=gError,
         cl.err=cl.err.loc,
         mean.cl.err=mean(cl.err.loc),
         ncl.err=ncl.err.loc,
         mean.ncl.err=mean(ncl.err.loc)
         )
}
```

```
##
## JAGS utilities
##
## Author: Pawel Gajer
## July 28, 2012
##

## return the mean and 95% CI
## modification of coda summary.mcmc()
## from output.R in coda/R
ci.mcmc <- function (object,short=TRUE,qs=NA, with.se=FALSE, n=NA) {
    x <- mcmc.list(object)

    if ( length(qs)==1 && is.na(qs) ) {
        if ( short && !with.se ) {
            qs <- c(0.5, 0.025, 0.975)
            statnames <- c("Mean", "Median", "2.5%", "97.5%")
        } else if (short && with.se) {
            qs <- c(0.5, 0.025, 0.975)
            statnames <- c("Mean", "S.E.", "Median", "2.5%", "97.5%")
        } else if (!short && !with.se) {
            qs <- c(0.5, 0.025, 0.975, 0.25, 0.75)
            statnames <- c("Mean", "Median", "2.5%", "97.5%", "25%", "75%")
        } else {
            qs <- c(0.5, 0.025, 0.975, 0.25, 0.75)
            statnames <- c("Mean", "S.E.", "Median", "2.5%", "97.5%", "25%", "75%")
        }
    } else {
        statnames <- c("Mean", paste(100*qs[1:length(qs)],"%",sep=""))
    }

    if ( !is.numeric(qs) )
        stop(paste("qs has to be a numeric vector; qs:",qs))

    if (is.matrix(x[[1]])) {
        xlong <- do.call("rbind", x)
    } else {
        xlong <- as.matrix(x)
    }

    nc <- ncol(xlong)

    xmean <- numeric(nc)
    for ( i in 1:nc )
        xmean[i] <- mean(xlong[,i],na.rm=TRUE)

    varquant <- matrix(nrow=nc,ncol=length(qs))
    for ( i in 1:nc )
        varquant[i,] <- quantile(xlong[,i],probs=qs,na.rm=TRUE)

    varstats <- matrix(nrow = nc, ncol = length(statnames),
                       dimnames = list(varnames(x), statnames))
    varstats[, 1] <- xmean
```

```
    if ( with.se ) {
        xsd <- numeric(nc)
        for ( i in 1:nc )
             xsd[i] <- sd(xlong[,i])/sqrt(n)

        varstats[, 2] <- xsd
        varstats[, 3:(length(qs)+2)] <- varquant

    } else {
        varstats[, 2:(length(qs)+1)] <- varquant
    }

    varstats <- drop(varstats)
    return(varstats)
}
```

```
##
## logistic regression mixed effects model
## i.e., ../3rd/logit_X_ri_hCauchy.bug
##
model {
    for( i in 1:length(y) ) {
        y[i] ~ dbern(p[i])
        logit(p[i]) <- mu + sum(a*X[i,]) + xi*eta[subjID[i]]
    }

    ## fixed effects
    prec <- 1.0E-5
    mu ~ dnorm(0.0, prec) # coefficient for type[i]=1

    for ( i in 1:nVar ) {
        a[i] ~ dnorm(0.0, prec)
    }

    ## random intercept
    xi ~ dnorm(0, tau.xi)
    tau.xi <- pow(25, -2)

    for ( i in 1:nSubj ) {
        eta[i] ~ dnorm(0.0, tau.eta)
    }
    tau.eta ~ dgamma(0.5, 0.5)
    sigma.eta <- abs(xi)/sqrt(tau.eta)
}
```

```
y <- factor(tt[rownames(pt), "Lac_Rh_ratio_category"])
levels(y)
```

```
## [1] "La/Rh ratio <= 0.05" "La/Rh ratio > 0.05"
```

```
r <- rf.cv2(pt[,1:20], y, subjID, nfolds=3, verbose=TRUE)
```

```
## [1] " i=1"
## [1] " i=2"
## [1] " i=3"
```

```
str(r)
```

```
## List of 18
##  $ cv.list     :List of 3
##   ..$ : int [1:24] 17 18 19 39 40 27 28 29 37 38 ...
##   ..$ : int [1:18] 41 42 36 21 51 12 13 16 32 31 ...
##   ..$ : int [1:17] 35 30 24 43 44 14 15 3 7 33 ...
##  $ y.pred.list :List of 3
##   ..$ : Factor w/ 2 levels "La/Rh ratio <= 0.05",..: 2 2 2 2 2 2 2 2 1 2 ...
##   .. ..- attr(*, "names")= chr [1:24] "S279879" "S279887" "S279895" "S280302" ...
##   ..$ : Factor w/ 2 levels "La/Rh ratio <= 0.05",..: 2 1 1 2 2 2 2 2 2 2 ...
##   .. ..- attr(*, "names")= chr [1:18] "S280323" "S280330" "S280267" "S279958" ...
##   ..$ : Factor w/ 2 levels "La/Rh ratio <= 0.05",..: 1 2 2 2 2 1 2 2 2 1 ...
##   .. ..- attr(*, "names")= chr [1:17] "S280239" "S280106" "S280011" "S280337" ...
##  $ error       :List of 3
##   ..$ : logi [1:24] TRUE TRUE TRUE FALSE TRUE FALSE ...
##   ..$ : logi [1:18] FALSE FALSE TRUE FALSE FALSE FALSE ...
##   ..$ : logi [1:17] TRUE FALSE FALSE FALSE FALSE FALSE ...
##  $ error.null  :List of 3
##   ..$ : logi [1:24] TRUE TRUE TRUE FALSE TRUE FALSE ...
##   ..$ : logi [1:18] FALSE TRUE FALSE FALSE FALSE FALSE ...
##   ..$ : logi [1:17] FALSE FALSE FALSE FALSE FALSE TRUE ...
##  $ sampleIdx   : int [1:59] 17 18 19 39 40 27 28 29 37 38 ...
##  $ imp         : num [1:20, 1:2] -0.002331 0 -0.005739 0.000519 0 ...
##   ..- attr(*, "dimnames")=List of 2
##   .. ..$ : chr [1:20] "f__Actinomycetaceae..g__Actinomyces" "f__Actinomycetaceae..g__
Varibaculum" "f__Corynebacteriaceae..g__Corynebacterium" "f__Dermabacteraceae..g__Dermab
acter" ...
##   .. ..$ : chr [1:2] "MeanDecreaseAccuracy" "MeanDecreaseGini"
##  $ rmse        : num 0
##  $ nmse        : num 0
##  $ mae         : num 0
##  $ nmae        : num 0
##  $ r2          : num 0
##  $ r2.pearson  : num 0
##  $ r2.spearman : num 0
##  $ gError      : num [1:59] 0 1 0 0 0 0 1 0 1 0 ...
##  $ cl.err      : num [1:3] 0.625 0.333 0.471
##  $ mean.cl.err : num 0.476
##  $ ncl.err     : num [1:3] 1 1 1.14
##  $ mean.ncl.err: num 1.05
```

```
## iterating 3-fold CV 10 times
nItr <- 10
imp <- matrix(0, nrow=ncol(pt), ncol=2)
cl.err <- c()
mean.cl.err <- c()
ncl.err <- c()
mean.ncl.err <- c()
for ( i in seq(nItr) ) {
    print(i)
    r <- rf.cv2(pt, y, subjID, nfolds=10, verbose=TRUE)
    imp <- imp + r$imp
    cl.err <- c(cl.err, r$cl.err)
    mean.cl.err <- c(mean.cl.err, r$mean.cl.err)
    ncl.err <- c(ncl.err, r$ncl.err)
    mean.ncl.err <- c(mean.ncl.err, r$mean.ncl.err)
}
```

```
## [1] 1
## [1] " i=1"
## [1] " i=2"
## [1] " i=3"
## [1] " i=4"
## [1] " i=5"
## [1] " i=6"
## [1] " i=7"
## [1] " i=8"
## [1] " i=9"
## [1] " i=10"
## [1] 2
## [1] " i=1"
## [1] " i=2"
## [1] " i=3"
## [1] " i=4"
## [1] " i=5"
## [1] " i=6"
## [1] " i=7"
## [1] " i=8"
## [1] " i=9"
## [1] " i=10"
## [1] 3
## [1] " i=1"
## [1] " i=2"
## [1] " i=3"
## [1] " i=4"
## [1] " i=5"
## [1] " i=6"
## [1] " i=7"
## [1] " i=8"
## [1] " i=9"
## [1] " i=10"
## [1] 4
## [1] " i=1"
## [1] " i=2"
## [1] " i=3"
## [1] " i=4"
## [1] " i=5"
## [1] " i=6"
## [1] " i=7"
## [1] " i=8"
## [1] " i=9"
## [1] " i=10"
## [1] 5
## [1] " i=1"
## [1] " i=2"
## [1] " i=3"
## [1] " i=4"
## [1] " i=5"
## [1] " i=6"
## [1] " i=7"
## [1] " i=8"
```

```
## [1] " i=9"
## [1] " i=10"
## [1] 6
## [1] " i=1"
## [1] " i=2"
## [1] " i=3"
## [1] " i=4"
## [1] " i=5"
## [1] " i=6"
## [1] " i=7"
## [1] " i=8"
## [1] " i=9"
## [1] " i=10"
## [1] 7
## [1] " i=1"
## [1] " i=2"
## [1] " i=3"
## [1] " i=4"
## [1] " i=5"
## [1] " i=6"
## [1] " i=7"
## [1] " i=8"
## [1] " i=9"
## [1] " i=10"
## [1] 8
## [1] " i=1"
## [1] " i=2"
## [1] " i=3"
## [1] " i=4"
## [1] " i=5"
## [1] " i=6"
## [1] " i=7"
## [1] " i=8"
## [1] " i=9"
## [1] " i=10"
## [1] 9
## [1] " i=1"
## [1] " i=2"
## [1] " i=3"
## [1] " i=4"
## [1] " i=5"
## [1] " i=6"
## [1] " i=7"
## [1] " i=8"
## [1] " i=9"
## [1] " i=10"
## [1] 10
## [1] " i=1"
## [1] " i=2"
## [1] " i=3"
## [1] " i=4"
## [1] " i=5"
## [1] " i=6"
## [1] " i=7"
```

```
## [1] " i=8"
## [1] " i=9"
## [1] " i=10"
```

```
imp <- imp/nItr
o <- order(imp[,2], decreasing=T)
imp <- imp[o,]
impPhs <- rownames(imp[1:15,])
```

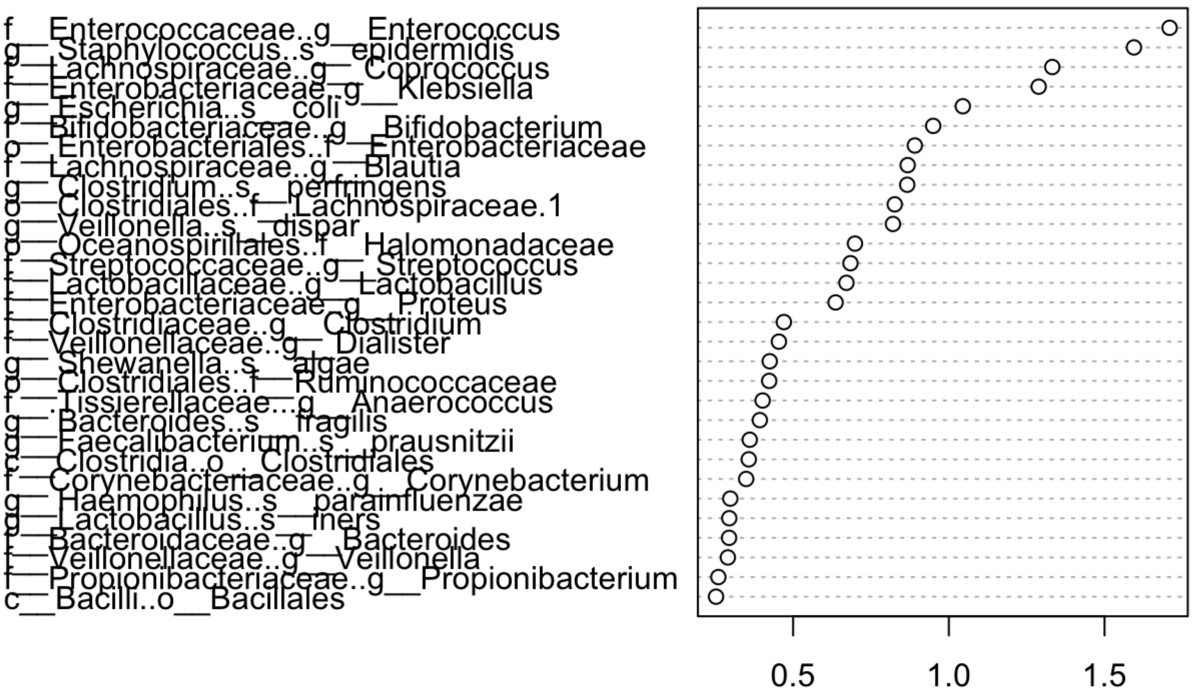

Variable Importance

```
## mixed effects logistic regression
yB <- ifelse(y=="La/Rh ratio > 0.05",1,0)
m <- glm( yB ~ pt[,impPhs[1:4]], family="binomial")

m <- list()
for ( i in 4:15 ) {
    m[[i]] <- glmer( y ~ scale(pt[,impPhs[1:i]]) + (1|subjID), family="binomial")
}

## Bayesian approach
selVar <- impPhs[1:15]
j.dat <- list(y=yB,
              X=scale(pt[,selVar]),
              nVar=length(selVar),
              subjID=as.integer(factor(subjID)),
              nSubj=length(unique(subjID)))

j.m <- jags.model("../3rd/logit_X_ri_hCauchy.bug", j.dat, n.chains=2)
```

```
## Compiling model graph
##    Resolving undeclared variables
##    Allocating nodes
## Graph information:
##    Observed stochastic nodes: 59
##    Unobserved stochastic nodes: 54
##    Total graph size: 1401
##
## Initializing model
```

```
nIter <- 10000
update(j.m, nIter)
j.pars <- c ("mu","a","sigma.eta")
j.out <- coda.samples(j.m, j.pars, thin=10, n.iter=nIter)
gelman.diag(j.out)
```

```
## Potential scale reduction factors:
##
##           Point est. Upper C.I.
## a[1]            1.00       1.01
## a[2]            1.01       1.01
## a[3]            1.00       1.01
## a[4]            1.00       1.00
## a[5]            1.00       1.00
## a[6]            1.01       1.01
## a[7]            1.02       1.08
## a[8]            1.00       1.00
## a[9]            1.00       1.01
## a[10]           1.00       1.01
## a[11]           1.00       1.00
## a[12]           1.02       1.04
## a[13]           1.01       1.02
## a[14]           1.01       1.03
## a[15]           1.01       1.04
## mu              1.01       1.01
## sigma.eta       1.00       1.00
##
## Multivariate psrf
##
## 1.03
```

```
j.ci <- ci.mcmc(j.out)

## p-values
pval <- c()
j.mat <- as.matrix(j.out)
nVar <- length(selVar)
j.a <- j.mat[,1:nVar]
for ( i in 1:nVar ) {
    if ( median(j.a[,i]) < 0 ){
        pval[i] <- 1 - pnorm(0, mean=median(j.a[,i]), sd=sd(j.a[,i]))
    } else {
        pval[i] <- pnorm(0, mean=median(j.a[,i]), sd=sd(j.a[,i]))
    }
}

j.tbl <- cbind(j.ci[1:15,c(1,3:4)], pval)
rownames(j.tbl) <- selVar
o <- order(j.tbl[,4])
(j.tbl <- j.tbl[o,])
```

```
##                                                   Mean      2.5%
## f__Lachnospiraceae..g__Coprococcus          -469.713234 -852.2636
## o__Clostridiales..f__Lachnospiraceae.1      -456.252340 -884.7643
## g__Veillonella..s__dispar                   -446.193722 -879.8985
## f__Bifidobacteriaceae..g__Bifidobacterium   -350.057207 -702.2972
## g__Clostridium..s__perfringens              -281.893723 -666.6840
## f__Enterobacteriaceae..g__Proteus           -354.735346 -866.8853
## g__Staphylococcus..s__epidermidis           -191.413041 -470.5548
## f__Lactobacillaceae..g__Lactobacillus       -142.066975 -407.3961
## g__Escherichia..s__coli                      118.239201 -196.0051
## o__Oceanospirillales..f__Halomonadaceae     -137.808298 -577.0737
## o__Enterobacteriales..f__Enterobacteriaceae   37.416424 -145.3739
## f__Enterococcaceae..g__Enterococcus           32.173068 -243.2429
## f__Streptococcaceae..g__Streptococcus        -29.486359 -393.3442
## f__Enterobacteriaceae..g__Klebsiella         -19.667224 -299.9117
## f__Lachnospiraceae..g__Blautia                -7.043833 -374.2506
##                                                  97.5%        pval
## f__Lachnospiraceae..g__Coprococcus          -139.45198 0.006524549
## o__Clostridiales..f__Lachnospiraceae.1      -148.17955 0.012699415
## g__Veillonella..s__dispar                   -118.75228 0.013673188
## f__Bifidobacteriaceae..g__Bifidobacterium   -109.04649 0.017093658
## g__Clostridium..s__perfringens                20.25681 0.070572567
## f__Enterobacteriaceae..g__Proteus             55.08462 0.075467888
## g__Staphylococcus..s__epidermidis             19.95198 0.086799398
## f__Lactobacillaceae..g__Lactobacillus         96.53359 0.130098494
## g__Escherichia..s__coli                      428.33157 0.226466157
## o__Oceanospirillales..f__Halomonadaceae      208.25710 0.271641020
## o__Enterobacteriales..f__Enterobacteriaceae  240.22072 0.372493479
## f__Enterococcaceae..g__Enterococcus          308.93976 0.417707443
## f__Streptococcaceae..g__Streptococcus        353.96027 0.425223563
## f__Enterobacteriaceae..g__Klebsiella         181.62770 0.478318873
## f__Lachnospiraceae..g__Blautia               326.14308 0.492598988
```
