# Supplementary material for: Microbial Biomarkers of Intestinal Barrier Maturation in Preterm Infants
Source: Front Microbiol. 2018 Nov 14;9:2755. doi: 10.3389/fmicb.2018.02755 (PMC6246636; doi:10.3389/fmicb.2018.02755)
Supplement: FIGURE S1 — Changes of intestinal permeability of each subject at study day 1, 8, and 15. Circle dot represents a sampling point, the line collecting points represents each subject at different time point. Different color of the lines specifies different subjects. The low and high intestinal permeability category was defined by a La/Rh > 0.05 or ≤0.05 respectively. [file Data_Sheet_1.zip › Supplementary_information/Legend_Supplementary.docx]

**Supplementary Figure S1.** Heatmap of the 50 most abundant intestinal bacterial taxa relative abundance in samples collected from 38 preterm infants enrolled in the study. The microbiota of 64 fecal samples were successfully characterized by high-throughput sequencing of the V3-V4 variable regions of 16S rRNA genes. The three sidebars indicate cluster, time, and intestinal permeability category, respectively. Ward linkage clustering was used to cluster samples based on their Jensen-Shannon distance calculated in vegan package in R (2). The samples with no IP assessment were included to generated the clusters. The low and high intestinal permeability category was defined by a La/Rh >0.05 or <=0.05 respectively (1). Taxonomic profiling of corresponding metagenomes further resolved *Klebsiella* spp. to *Klebsiella pneumoniae*, *Enterococcus spp.* to *Enterococcus faecalis*, and *Bifidobacterium* *spp.* to *Bifidobacterium breve*.

**Supplementary Figure S2**. Prediction and residual plots of balance tree analysis. (**A**) The projected residuals of input and prediction onto the plotted balances. The predicted points lie within the same region as the original communities, indicating a high credibility of the prediction. (**B**) The residuals have roughly the same variance as the predictions and are mostly within ±2. The top two balances are plotted, and the percentage of variance described by the plotted balances is indicated on the axes.

**Supplementary Figure S3**. Comparison of the relative abundance of members of *Clostridiales* (**A**), *Bifidobacteriales* (**B**), *Staphylococcaceae* (**C**), and *Enterobacteriaceae* (**D**) on phase I (1-15 days old) and III (6-18 months old) infants (4). Bars represent the relative abundance of each bacterial group in each sample. Dotted line represents mean, solid line represents median relative abundance. The alpha value for the non-parametric factorial Kruskal-Wallis sum-rank test was 0.05 and the threshold for the logarithmic LDA model (3) score for discriminative features was set at 2.0.

**Supplementary Figure S4**. Dependence between intestinal permeability binary index and the log relative abundance of *Coprococcus* (**A**) and *Clostridiales* (**B)**. An adaptive spline logistic regression model implemented in spmrf R package was applied to the phylotypes present in at least 15% of all samples. Low IP: La/Rh < 0.05; high IP: La/Rh >= 0.05. Bar on x-axis indicates the dichotomous categories in intestinal permeability: gray: low IP; red: high IP.

**Supplementary Figure S5**. Mean decrease Gini index random forest importance measure for the classification of intestinal permeability using relative abundance of bacterial phylotypes. Top 30 measurements were shown on the figure, and the top 15 phylotypes were used to fit a random effect logistic regression model of IP. Measurement is calculated using random forest supervised machine learning scheme implemented in R package randomForest (48). Low IP: La/Rh < 0.05; high IP: La/Rh >= 0.05.

**Supplementary Figure S6**. Correlation plot between the bacterial species that are most correlated in normalized transcriptome profiles. Correlation plots were generated using *corrplot* package (58) in R. Spearman correlation coefficients are shown in figure. Y and x-axis indicate the relative abundance on logarithm 10 scale of the bacterium labelled.

**Supplementary Figure S7**. Dependence between time reaching full breastmilk feeding and the log relative abundance of *Clostridiales* (**A**) and *Klebsiella* (**B**). An adaptive spline logistic regression model implemented in spmrf R package was applied to the phylotypes present in at least 15% of all samples. More than 10 postnatal days to reach full breastmilk feeding is considered late time, while less than 10 postnatal days is considered early time. Bayesian goodness-of-fit p-value implemented in R package rstan ^63^ was used to access the significance of the association between phylotypes and investigated factors. Bar on x-axis indicates the dichotomous categories in time point reaching full breast milk feeding: gray: earlier time (<10 days); red: late time (>=10 days).

**Supplementary Figure S8**. Dependence between duration of antibiotic treatment and the log relative abundance of *Clostridiales* (**A**) and *Lachnospiraceae* (**B**). An adaptive spline logistic regression model implemented in spmrf R package was applied to the phylotypes present in at least 15% of all samples. Use of antibiotics for more than 4 days was considered long duration, while less or equal to 4 days was considered short duration. Bayesian goodness-of-fit p-value implemented in R package rstan ^63^ was used to access the significance of the association between phylotypes and investigated factors. Bar on x-axis indicates the dichotomous categories in antibiotic treatment duration: gray: shorter duration (<4 days); red: longer duration (>=4 days).

**Supplementary File S1**. R markdown code implementation of the adaptive spline logistic regression model using the *spmrf* R package (46).

**Supplementary File S2**. R markdown code implemention of the random forest supervised machine learning scheme using the R package randomForest (48).

**Supplementary File S3**. Taxonomic table and metadata used in this study. (A) The demographic, obstetric, and neonatal characteristics for each subject included in this study and the phylotype using high-throughput sequencing of the V3-V4 variable regions of 16S rRNA genes. (B) Taxonomic profiles of stool samples from 16 infants at 6-24 months (Phase II/III) of age born at term, using high-throughput sequencing of the V3-V4 variable regions of 16S rRNA genes. (C) Taxonomic profiles of 19 stool samples, using metagenome sequencing and profiled using MetaPhlAn version 2 (Segata et al., 2012).

**Supplementary File S4**. Statistics summary of Gneiss balance tree analysis using the simplicial linear regression (Morton et al., 2017). Covariates of antibiotics use, maternal antibiotics use, delivery mode, preterm premature rupture of membranes (pPROM), feeding pattern, IP, birthweight, gender, ethnicity, gestational age (GA) and postmenstrual age (PMA) were included in the analysis.

**Supplementary File S5**. Statistics summary of random effect logistic regression model of intestinal permeability and associations with relative abundance of bacterial phylotypes. The top 15 phylotypes with the highest mean decrease gini index importance measure listed in Supplemental Figure 5 were included in this analysis.
