# Supplementary material for: Microbial Biomarkers of Intestinal Barrier Maturation in Preterm Infants
Source: Front Microbiol. 2018 Nov 14;9:2755. doi: 10.3389/fmicb.2018.02755 (PMC6246636; doi:10.3389/fmicb.2018.02755)
Supplement: FIGURE S1 — Changes of intestinal permeability of each subject at study day 1, 8, and 15. Circle dot represents a sampling point, the line collecting points represents each subject at different time point. Different color of the lines specifies different subjects. The low and high intestinal permeability category was defined by a La/Rh > 0.05 or ≤0.05 respectively. [file Data_Sheet_1.zip › Supplementary_information/Supplemental_figure/Supplementary Figure S5.pdf]

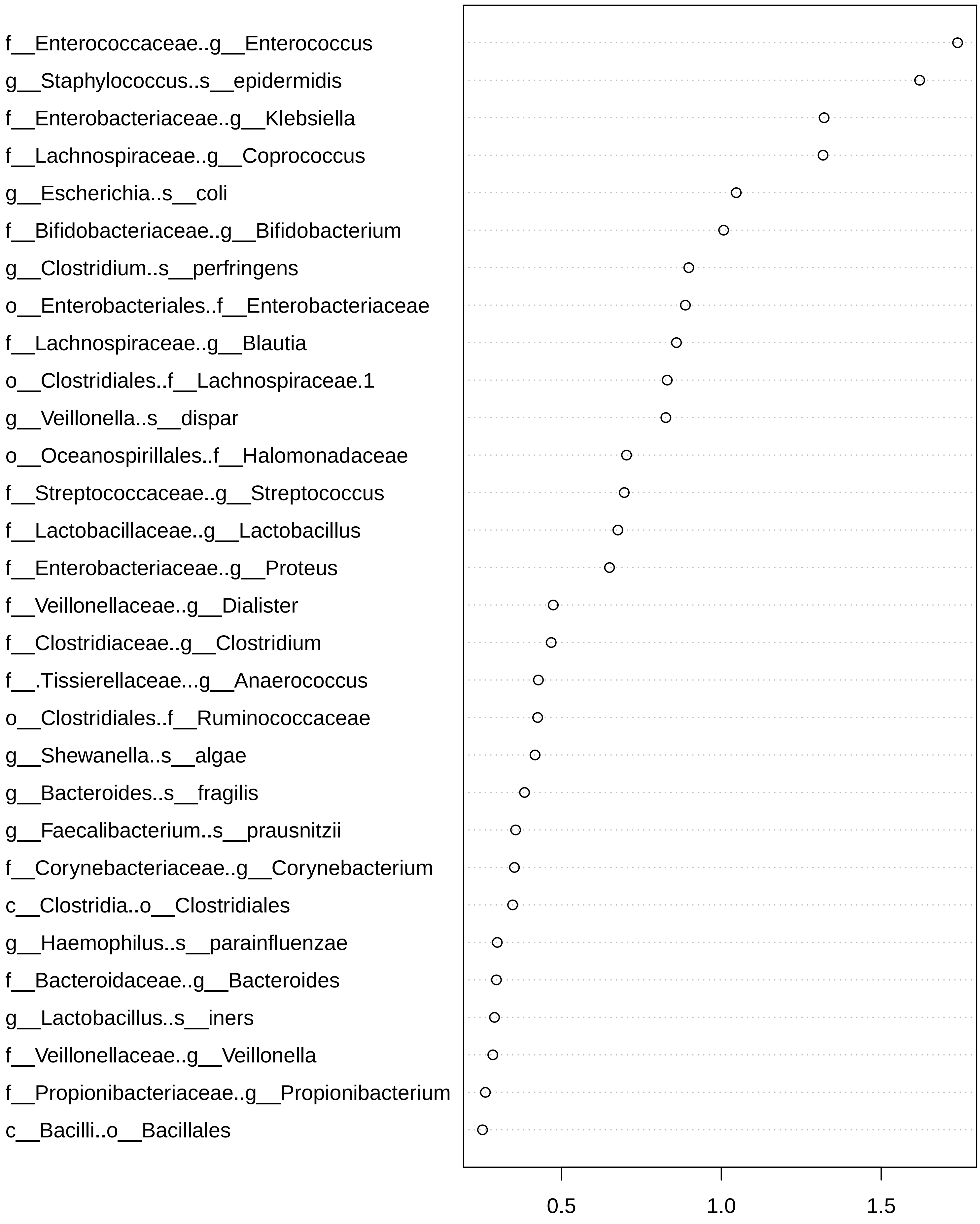
f__Enterococcaceae..g__Enterococcus
g__Staphylococcus..s__epidermidis
f__Enterobacteriaceae..g__Klebsiella
f__Lachnospiraceae..g__Coprococcus
g__Escherichia..s__coli
f__Bifidobacteriaceae..g__Bifidobacterium
g__Clostridium..s__perfringens
o__Enterobacteriales..f__Enterobacteriaceae
f__Lachnospiraceae..g__Blautia
o__Clostridiales..f__Lachnospiraceae.1
g__Veillonella..s__dispar
o__Oceanospirillales..f__Halomonadaceae
f__Streptococcaceae..g__Streptococcus
f__Lactobacillaceae..g__Lactobacillus
f__Enterobacteriaceae..g__Proteus
f__Veillonellaceae..g__Dialister
f__Clostridiaceae..g__Clostridium
f__.Tissierellaceae...g__Anaerococcus
o__Clostridiales..f__Ruminococcaceae
g__Shewanella..s__algae
g__Bacteroides..s__fragilis
g__Faecalibacterium..s__prausnitzii
f__Corynebacteriaceae..g__Corynebacterium
c__Clostridia..o__Clostridiales
g__Haemophilus..s__parainfluenzae
f__Bacteroidaceae..g__Bacteroides
g__Lactobacillus..s__iners
f__Veillonellaceae..g__Veillonella
f__Propionibacteriaceae..g__Propionibacterium
c__Bacilli..o__Bacillales
0.5
1.0
1.5
