# Supplementary material for: Microbial Biomarkers of Intestinal Barrier Maturation in Preterm Infants
Source: Front Microbiol. 2018 Nov 14;9:2755. doi: 10.3389/fmicb.2018.02755 (PMC6246636; doi:10.3389/fmicb.2018.02755)
Supplement: FIGURE S1 — Changes of intestinal permeability of each subject at study day 1, 8, and 15. Circle dot represents a sampling point, the line collecting points represents each subject at different time point. Different color of the lines specifies different subjects. The low and high intestinal permeability category was defined by a La/Rh > 0.05 or ≤0.05 respectively. [file Data_Sheet_1.zip › Supplementary_information/Supplemental_figure/Supplementary Figure S3.pdf]

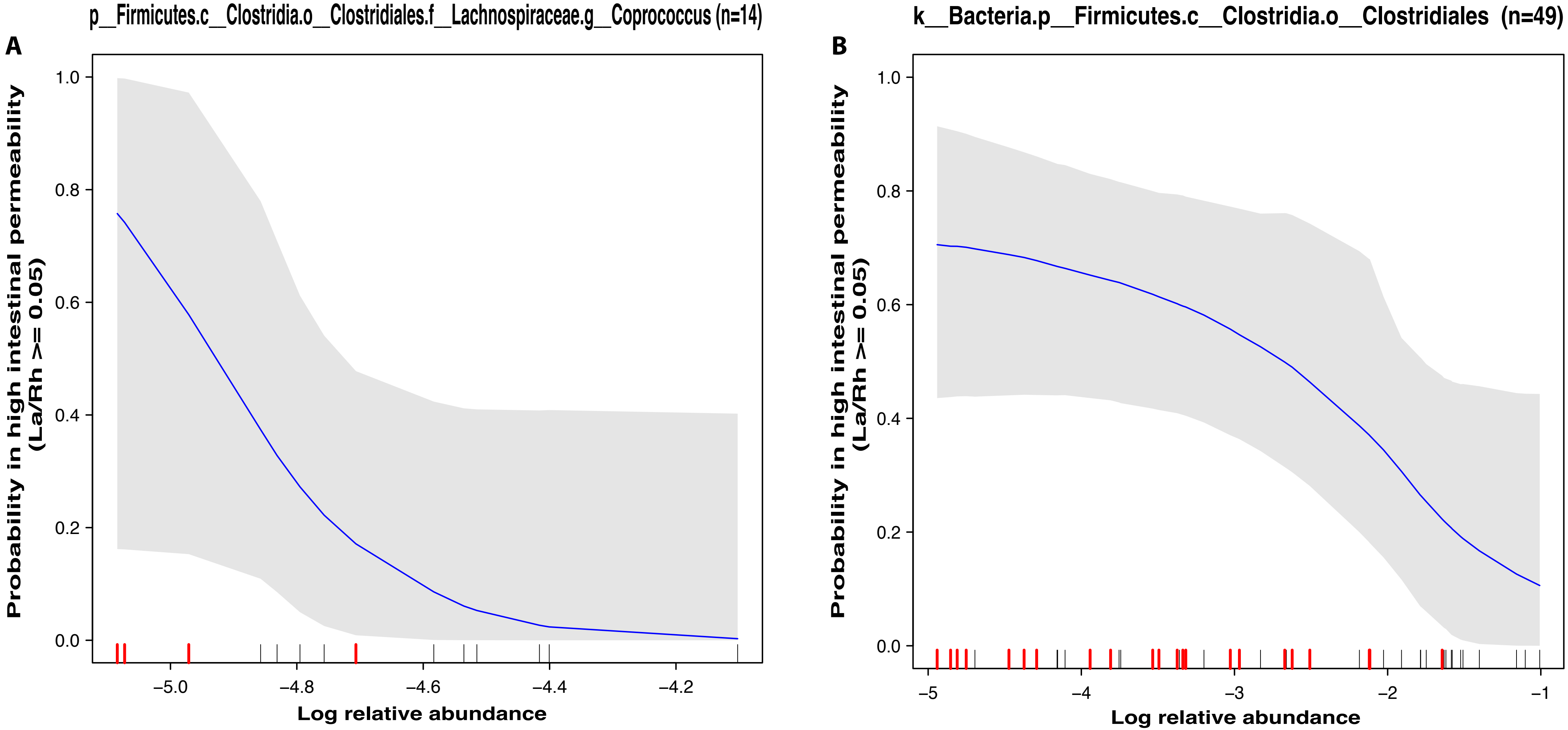
p__Firmicutes.c__Clostridia.o__Clostridiales.f__Lachnospiraceae.g__Coprococcus (n=14)
A
Probability in high intestinal permeability (La/Rh >= 0.05)
1.0
0.8
0.6
0.4
0.2
0.0
−5.0
−4.8
−4.6
−4.4
−4.2
Log relative abundance
k__Bacteria.p__Firmicutes.c__Clostridia.o__Clostridiales (n=49)
B
Probability in high intestinal permeability (La/Rh >= 0.05)
1.0
0.8
0.6
0.4
0.2
0.0
−5
−4
−3
−2
−1
Log relative abundance
